# Diversity and Evolutionary Dynamics of Antiphage Defense Systems in *Ralstonia solanacearum* Species Complex

**DOI:** 10.3389/fmicb.2020.00961

**Published:** 2020-05-20

**Authors:** José A. Castillo, Henry Secaira-Morocho, Stephanie Maldonado, Katlheen N. Sarmiento

**Affiliations:** School of Biological Sciences and Engineering, Yachay Tech University, San Miguel de Urcuquí, Ecuador

**Keywords:** plant pathogenic bacteria, microbial defense system, bacteria-phage co-evolution, foreign DNA, defense islands

## Abstract

Over the years, many researchers have reported a great diversity of bacteriophages infecting members of the *Ralstonia solanacearum* species complex (RSSC). This diversity has driven bacterial evolution by leading the emergence and maintenance of bacterial defense systems to combat phage infection. In this work, we present an *in silico* study of the arsenal of defense systems that RSSC harbors and their evolutionary history. For this purpose, we used a combination of genomic, phylogenetic and associative methods. We found that in addition to the CRISPR-Cas system already reported, there are eight other antiphage defense systems including the well-known Restriction-Modification and Toxin-Antitoxin systems. Furthermore, we found a tenth defense system, which is dedicated to reducing the incidence of plasmid transformation in bacteria. We undertook an analysis of the gene gain and loss patterns of the defense systems in 15 genomes of RSSC. Results indicate that the dynamics are inclined toward the gain of defense genes as opposed to the rest of the genes that were preferably lost throughout evolution. This was confirmed by evidence on independent gene acquisition that has occurred by profuse horizontal transfer. The mutation and recombination rates were calculated as a proxy of evolutionary rates. Again, genes encoding the defense systems follow different rates of evolution respect to the rest of the genes. These results lead us to conclude that the evolution of RSSC defense systems is highly dynamic and responds to a different evolutionary regime than the rest of the genes in the genomes of RSSC.

## Introduction

The dynamic interaction between bacteria and bacteriophages (phages henceforth) drives microbial evolution. This process leads to the rapid evolution of defense systems to combat phage infection and parasitism. The bacterial world shows complex and abundant mechanisms of defense encoded in bacterial and archaeal genomes; however, there are also phage mechanisms to counteract bacterial defense systems (Rostøl and Marraffini, 2019). Apart from that, some defense systems are mediated by the phages, once the lysogeny is established efficiently, the prophage-expressed genes strongly inhibit lytic infection of the same or related phages (Montgomery et al., 2019).

The bacterial defense mechanisms act to prevent all stages of phage infection and spreading inside the host. These multiple defense strategies include surface modifications to prevent adsorption of phages (Dy et al., 2014a), restriction–modification mechanism that degrade phage DNA and modify the bacterial genome (Tock and Dryden, 2005), abortive infection that results in the death of the infected bacteria which limits phage spread via an ‘altruistic suicide’ (Dy et al., 2014b), CRISPR–Cas system that is an ‘adaptive immunity’ (Marraffini, 2015), Argonaute system that uses a DNA or RNA molecule as a guide to silence phage DNA by nucleolytic cleavage (Swarts et al., 2014). Recently, new systems that have been discovered, although the molecular mechanism of action is not known in detail, have demonstrated high phage controlling power and broad distribution in bacteria and archaea (Doron et al., 2018).

Bacterial defense systems are under constant selective pressure by phage attack. The bacteria-phage competitive relationship drives bacteria to maintain or acquire a relatively adequate fitness, sufficient to inhibit phage infection and proliferation. This evolutionary process generates a great diversity, which implies different gene gain and loss dynamics. Of these two genome dynamic events, gene loss is common in bacterial genomes and, on the contrary, gene gain is an incidental event (Wolf and Koonin, 2013). The most common mechanism for gene gain is horizontal gene transfer (HGT) which results in genome expansion and acquisition of new functions (Ochman et al., 2000). Alternatively, gene duplication also generates greater availability of diverse genes that are useful to face new challenges usually imposed by the diversified phage attack (Zhang, 2003). The rate of gene gain and loss also differs mostly depending on the biological activities of the genes. The most stable are the genes that are devoted to basic or essential cell processes such as translation during protein synthesis (Puigbò et al., 2014). In the case of defense systems, they generally show 3 times more gene loss than gain and an order of magnitude more common than the duplication of gene families (Puigbò et al., 2017).

*Ralstonia solanacearum* species complex (RSSC) is a diverse group of bacterial pathogens that infect and cause diseases in dozens of plant families. Members of this complex are the causal agent of bacterial wilt mainly in Solanaceae family of plants, Moko disease of banana and brown rot of potato (Peeters et al., 2013). It is considered a major pathogen since it heavily affects agricultural production worldwide (Mansfield et al., 2012). The diversity of RSSC allowed classification of four groups called phylotypes, of which, the phylotype II was subdivided into two subgroups (IIA and IIB) (Fegan and Prior, 2005). However, the current taxonomic classification of RSSC comprises three different species: *R. pseudosolanacearum* (which includes phylotypes I and III), *R. solanacearum* (phylotype II) and *R. syzygii* (phylotype IV, the original *R. syzygii* and the blood disease bacterium) (Safni et al., 2014; Prior et al., 2016).

*Ralstonia solanacearum* species complex is mostly a soil-borne pathogen although insect vectors also transmit some particular subgroups to host plants. RSSC first invades plant roots through wounds or natural openings, colonizes the root intercellular spaces and then invades xylem vessels eventually leading the plant host to death (Hikichi et al., 2017). This dual lifestyle (soil-plant or insect-plant) has placed RSSC at a high risk of phage attack. Certainly, over the years many researchers have been reporting a large number of phages infecting RSSC and these spans a considerable large range of genetic diversity (Table 1). Three are the main viral families that attack RSSC: Inoviridae, Myoviridae, and Podoviridae, however a member of a fourth family has recently been found: phage ϕRS138 that belongs to family Siphoviridae (Van Truong Thi et al., 2016). Depending on the family to which phages belong, they contain single- or double-stranded DNA genomes and are filamentous or showing a head-tail structure. Most interesting, many phages are lytic which opens the possibility to use them in phage therapy to control different strains of RSSC.

**TABLE 1:** Phages infecting RSSC.

**Phage name**	**Capsid type**	**Viral replication**	**Family**	**Genome type**	**Genome size (bp)**	**References**
PE226	Filamentous	Lytic	Inoviridae	ssDNA	5,475	Murugaiyan et al., 2011
φRS603	Filamentous	Lytic	Inoviridae	ssDNA	7,679	Van et al., 2014
φRSA1	Head-Tail	Lytic	Myoviridae	dsDNA	38,760	Fujiwara et al., 2008
φRSB1	Head-Tail	Lytic	Podoviridae	dsDNA	43,079	Kawasaki et al., 2009
φRSB2	Head-Tail	Lytic	Podoviridae	dsDNA	40,411	Kawasaki et al., 2016
φRSB3	Head-Tail	Lytic	Podoviridae	dsDNA	44,578	Kawasaki et al., 2016
φRSJ2	Head-Tail	Lytic	Podoviridae	dsDNA	44,684	Kawasaki et al., 2016
φRSJ5	Head-Tail	Lytic	Podoviridae	dsDNA	44,067	Kawasaki et al., 2016
RSK1	Head-Tail	Lytic	Podoviridae	dsDNA	40,471	Kotera et al., unpublished*^*a*^*
DU_RP_II	Head-Tail	Lytic	Podoviridae	dsDNA	42,091	Park, 2018
φRSL1	Head-Tail	Lytic	Myoviridae	dsDNA	231,255	Yamada et al., 2010
φRSM1	Filamentous	Lytic	Inoviridae	ssDNA	8,999	Kawasaki et al., 2007
φRSM3	Filamentous	Prophage	Inoviridae	ssDNA	8,929	Askora et al., 2009
φRSM4	Filamentous	Prophage	Inoviridae	ssDNA	7,929	Askora et al., 2009
RSMSuper	Filamentous	ND	Inoviridae	ssDNA	8,956	Askora et al., unpublished*^*b*^*
φRS611	Filamentous	ND	Inoviridae	ssDNA	6,386	Van et al., 2015
φRSS0	Filamentous	Prophage and free	Inoviridae	ssDNA	7,288	Addy et al., 2018, unpublished*^*c*^*
φRSS1	Filamentous	Lytic	Inoviridae	ssDNA	6,662	Kawasaki et al., 2007
RSS30	Filamentous	ND	Inoviridae	ssDNA	8,576	Kawasaki et al., unpublished*^*d*^*
RSY1	Head-Tail	Lysogenic	Myoviridae	dsDNA	40,002	Askora et al., 2017
RsoP1IDN	Head-Tail	Lytic	Podoviridae	dsDNA	41,135	Addy et al., 2018
RsoP1EGY	Head-Tail	Lytic	Podoviridae	dsDNA	41,297	Ahmad et al., 2018
RPSC1	Head-Tail	Lytic	Podoviridae	dsDNA	39,628	Liao, 2018
φRS138	Head-Tail	Lytic	Siphoviridae	dsDNA	41,941	Van Truong Thi et al., 2016
RSL2	Head-Tail	Lytic	Myoviridae	dsDNA	223,932	Bhunchoth et al., 2016
RSF1	Head-Tail	Lytic	Myoviridae	dsDNA	222,888	Bhunchoth et al., 2016
φRs551	Filamentous	Lytic, prophage	Inoviridae	ssDNA	7,929	Ahmad et al., 2017
RSPI1	Head-Tail	Lytic	Podoviridae	dsDNA	43,211	Su et al., 2017
PE204	Head-Tail	Lytic	Podoviridae	dsDNA	21,000	Bae, 2012
P4282	Head-Tail	Lytic	Podoviridae	dsDNA	39,300	Ozawa et al., 2001
φAP1	Head-Tail	Lytic	Podoviridae	dsDNA	44,793	da Silva, Xavier et al., 2018
RsoM1USA	Head-Tail	Lytic	Myoviridae	dsDNA	39,309	Addy et al., 2019
GP4	Head-Tail	Lytic	Podoviridae	dsDNA	61,129	Wang R. et al., 2019
vRsoP-WF2, WM2, WR2	Head-Tail	Lytic	Podoviridae	dsDNA	40,409 40,861 40,408	Álvarez et al., 2019

*ND, no data available. *^*a*^*https://www.ncbi.nlm.nih.gov/nuccore/NC_022915. *^*b*^*https://www.ncbi.nlm.nih.gov/nuccore/AB981170.1, *^*c*^*https://www.ncbi.nlm.nih.gov/nuccore/NC_019548, *^*d*^*https://www.ncbi.nlm.nih.gov/nuccore/NC_021862.*

The RSSC-phage relationship implies that this bacterial group has had to evolve to acquire and update a repertoire of defense systems while phages adapt to overcome these mechanisms. This competitive interaction has created an evolutionary arms race that has driven the production of the extraordinary diversity of bacterial defense mechanisms in RSSC to hinder phage aggressions. However, the relative abundance, diversity, and evolution of the defense systems that RSSC possesses are unknown, with the exception of the CRISPR system reported by da Silva, Xavier et al. (2019). Therefore, in this study, we present a detailed study of the arsenal of defense systems that RSSC harbors and their evolutionary dynamics. For this purpose, we used a combination of genomic, phylogenetic and associative methods to determine the diversity of defense systems, to analyze the gene gain/loss dynamics, and to measure the evolutionary rate to compare to non-defense genes in the RSSC genomes.

The study of defense systems in RSSC takes greater relevance in the context of biological control against bacterial wilt. It is urgent to apply effective control strategies, which may include the use and application of phages. Lytic phages are of greater interest since they proliferate and destroy the host bacterial cell. Thus, phage therapy is a promising strategy against bacterial wilt since there are already some reported successful cases in the control of this serious disease using phages (Fujiwara et al., 2011; Wang X. et al., 2019; Álvarez et al., 2019).

## Materials and Methods

### Sequence Data and Protein Family Assignment

We searched for defense systems presence/absence in all RSSC genomic sequences available in databases to date (June 2019); however, we focused to perform the evolutionary analyses in only fifteen strains that correspond to reference genomes for the four phylotypes of RSSC. The fifteen full-genome sequences were selected to maximize diversity in terms of phylotypes and sequevars (sequevar is a subdivision based on the endoglucanase gene sequence, Fegan and Prior, 2005), and were downloaded from NCBI’s FTP server^1^. Due to the scarcity of available genomic sequences of phylotype III (only 3), we rather work with an even number of sequences (3 sequences) for each phylotype (including phylotypes IIA and IIB), totaling the 15 genome sequences (see Supplementary Table 1 for strains and genomic accession numbers).

We searched protein sequence homology in whole RSSC genomes using the HMMER online tool^2^ (Finn et al., 2011). This search allowed us to find Pfam (El-Gebali et al., 2019) accessions for the proteins encoded in the genomes. Similarly, for Clusters of Orthologous Groups (COGs), we used the online server Batch CD-Search tool^3^ (which is useful for both a conserved domain search on multiple protein sequences and for COG designation.

A list of Pfam accessions involved in bacterial defense systems was constructed (see Table 2) using the information of different articles that report experimental results. We complemented this data with a list of COG accessions related to antiphage defense, if available. Defense proteins in RSSC genomes were identified based on the list of known Pfams and COGs involved on defense using the complete set of Pfam obtained from RSSC genomes using the HMMER online tool as said above. For some defense systems, we used additional tools: to detect CRISPR genes, we visited CRISPRCasFinder online service^4^ and the CRISPI Interactive database^5^; to identify TA genes, we reviewed genome annotations gb files; to detect RM genes, we searched the REBASE^6^, a database of restriction enzymes and related proteins. To construct the Venn diagram, we employed an online tool available at http://bioinformatics.psb.ugent.be/webtools/Venn/.

**TABLE 2:** Antiphage and anti-plasmid defense systems in RSSC.

**Protein family/domain**	**References**	**Defense system*^*a*^***	**Annotation/Function**	**Phylotypes*^*b*^***				
				**I**	**IIA**	**IIB**	**III**	**IV**
PF18019/COG1203	Makarova et al., 2011	CRISPR	CRISPR-associated helicase Cas3	0(1)	1	1	1	0(1)
PF01867/COG1518	Makarova et al., 2011	CRISPR	CRISPR associated protein Cas1	0(1)	1	1	1	0(1)
PF08798	Makarova et al., 2011	CRISPR	CRISPR associated protein Cas6/Cse3/CasE	0(1)	1	1	1	0(1)
PF09481	Makarova et al., 2011	CRISPR	CRISPR-associated protein Cse1/CasA	0(1)	1	1	1	0(1)
PF09485	Makarova et al., 2011	CRISPR	CRISPR-associated protein Cse2/CasB	0(1)	1	1	1	0(1)
PF09704	Makarova et al., 2011	CRISPR	CRISPR-associated protein Cas5/CasD	0(1)	1	1	1	0(1)
PF09707	Makarova et al., 2011	CRISPR	CRISPR-associated protein Cas2	0(1)	1	1	1	0(1)
PF09344	Makarova et al., 2011	CRISPR	CRISPR-associated protein Cas7/Cse4/CasC, CT1975-like protein	0(1)	1	1	1	0(1)
PF13175/COG1106, COG3593, COG4938	Doron et al., 2018	Gabija	GajA, ATPase domain. Predicted ATP dependent endonuclease.	1^∗^	1	1^∗^	1^∗^	1
PF13361, PF00580, PF13245/COG0210	Doron et al., 2018	Gabija	GajB, UvrD-like helicase C-terminal domain. AAA domain.	1^∗^	1	1^∗^	1^∗^	1
PF08878	Doron et al., 2018	Hachiman	HamA, unknown function	0	0	0	0	1
PF00270, PF00271/COG1204, COG0553	Doron et al., 2018	Hachiman	HamB, replicative Superfamily II DNA or RNA helicase, SNF2 family.	1	1	1	1	1
PF13289	Doron et al., 2018	Thoeris	ThsA, Sir2/Macro domain (NAD binding)	1	0	1	1^∗^	1^∗^
PF13676	Doron et al., 2018	Thoeris	ThsB, TIR domain, Toll-like receptors family.	1	0	1	0	1^∗^
PF14236	Doron et al., 2018	Druantia, type I	DruA, unknown function, DUF4338 domain.	(1)	0	(1)	0	(1)
No family/domain annotation	Doron et al., 2018	Druantia, type I	DruB, unknown function	0	(1)	(1)	0	0
No family/domain annotation	Doron et al., 2018	Druantia, type I	DruC, unknown function	(1)	(1)	(1)	0	0
No family/domain annotation	Doron et al., 2018	Druantia, type I	DruD, unknown function	0	0	(1)	0	0
PF09369/COG1205	Doron et al., 2018	Druantia type I	DruE, Replicative superfamily II, ATP-dependent helicase, C-terminal Zn-binding domain, DUF1998 domain.	(1)	(1)	(1)	1	(1)
PF09660	Doron et al., 2018	Wadjet	JetA, unknown function, MukF-like.	1(1)	(1)	1(1)	1	1
PF09661	Doron et al., 2018	Wadjet	JetB, unknown function, DUF4194 domain, MukE-like.	1(1)	(1)	1(1)	1	1
PF02463/COG0419, COG1196	Doron et al., 2018	Wadjet	JetC, ATPase N terminal domain, MukB-like, chromosome segregation ATPase, ATP binding domain, DNA repair exonuclease	1(1)	1(1)	1(1)	1	1
PF09664, PF11796, PF09983	Doron et al., 2018	Wadjet	JetD, unknown function, Topoisomerase VI predicted by structural similarities	1(1)	(1)	1(1)	1	1
PF04380	Ryazansky et al., 2018	Argonaute	nuclease PIWI domain	(1)	1	1^∗^	1^∗^	1^∗^
PF13289	Ryazansky et al., 2018	Argonaute	SIR2 family protein	(1)	0	1^∗^	1^∗^	1^∗^
PF00145/COG0270	Makarova et al., 2011; Doron et al., 2018	RM	C-5 cytosine-specific DNA methylase, DNA methyltransferase	1	0	0	1	1
PF01555/COG0863, COG2189	Makarova et al., 2011; Doron et al., 2018	RM	DNA methyltransferase, Adenine-specific methylase	1	1	1	1	1
PF01844/COG1403	Makarova et al., 2011; Doron et al., 2018	RM	Restriction endonuclease, McrA/HNH family	1	1	1	1	1
PF02384/COG0286	Makarova et al., 2011; Doron et al., 2018	RM	N-6 DNA Methylase. Type I restriction-modification system, DNA methylase subunit.	1	1	1	1	1
PF04851/COG1061, COG1201	Makarova et al., 2011; Doron et al., 2018	RM	Type III restriction enzyme, res subunit. DNA or RNA helicase superfamily II. Lhr-like helicase.	1	1	1	1	1
PF08463/COG4096	Makarova et al., 2011; Doron et al., 2018	RM	EcoEI R protein C-terminal., Site-specific restriction-modification system, R subunit or related helicase	1	1	1	1	1
PF10544	Makarova et al., 2011; Doron et al., 2018	RM	Type III restriction enzyme, res subunit. T5orf172 domain.	0	1	0	0	0
PF10593	Makarova et al., 2011; Doron et al., 2018	RM	Z1 domain	1	0	0	0	1
PF12161	Makarova et al., 2011; Doron et al., 2018	RM	N-terminal domain the methylase subunit of Type I DNA methyltransferases	1	0	0	1	1
PF13156	Makarova et al., 2011; Doron et al., 2018	RM	Type IIG protein, Restriction endonuclease	0	0	1	0	0
PF01170	Makarova et al., 2011; Doron et al., 2018	RM	Putative RNA methylase family UPF0020	1	1	1	1	1
PF01420	Makarova et al., 2011; Doron et al., 2018	RM	Methylase_S, Type I restriction modification DNA specificity domain	0	0	0	1	1
PF03852/COG2852	Makarova et al., 2011; Doron et al., 2018	RM	Very short patch repair (VSP)	1	0	0	1	1
PF04313	Makarova et al., 2011; Doron et al., 2018	RM	Type I restriction enzyme R protein N terminus, HSDR_N	1	1	0	1	1
PF11867	Makarova et al., 2011; Doron et al., 2018	RM	DUF3387 domain	0	0	0	1	0
PF14511	Makarova et al., 2011; Doron et al., 2018	RM	Type II restriction endonuclease *Eco*O109I	0	0	0	1	0
PF18766	Makarova et al., 2011; Doron et al., 2018	RM	SWI2_SNF2 ATPase	0	1	1	1	1
PF04471/COG1715, COG1787	Makarova et al., 2011; Doron et al., 2018	RM	Restriction endonuclease Mrr	1	1	1	1	1
PF08843/COG2253, COG4849	Makarova et al., 2011; Doron et al., 2018	TA, Abi	Nucleotidyl transferase AbiEii toxin, Type IV TA system.	1	1	1	1	1
PF09952/COG4861	Makarova et al., 2011; Doron et al., 2018	TA, Abi	Transcriptional regulator, AbiEi antitoxin, Type IV TA system. Putative toxin has a nucleotidyl transferase domain.	1	1	1	1	1
PF17194	Makarova et al., 2011; Doron et al., 2018	TA, Abi	Transcriptional regulator, AbiEi antitoxin N-terminal domain	1	0	0	1	1
PF11459	Makarova et al., 2011; Doron et al., 2018	TA, Abi	Transcriptional regulator, AbiEi antitoxin, Type IV TA system	1	0	0	1	1
PF13338	Makarova et al., 2011; Doron et al., 2018	TA, Abi	Transcriptional regulator, AbiEi antitoxin	0	1	0	1	1
PF01381	Sberro et al., 2013	TA	helix-turn-helix (HTH) motif capable of binding DNA	1	1	1	1	1
PF01850	Sberro et al., 2013	TA	PIN domain with nuclease activity to cleave single stranded RNA	1	1	1	1	1
PF02604	Sberro et al., 2013	TA	Antitoxin Phd_YefM, type II toxin-antitoxin system	1	1	1	1	1
PF02794	Sberro et al., 2013	TA	RTX toxin acyltransferase family	1	1	1	1	1
PF03364	Sberro et al., 2013	TA	Polyketide cyclase/dehydrase and lipid transport	1	1	1	1	1
PF03658	Sberro et al., 2013	TA	RnfH family Ubiquitin	1	1	1	1	1
PF03693	Sberro et al., 2013	TA	Bacterial antitoxin of ParD toxin-antitoxin type II system and RHH	1	0	1	1	1
PF04014	Sberro et al., 2013	TA	Antidote-toxin recognition MazE, bacterial antitoxin	1	1	1	1	0
PF04221	Sberro et al., 2013	TA	RelB antitoxin	0	1	1	0	0
PF05015	Sberro et al., 2013	TA	RelE-like toxin of type II toxin-antitoxin system HigB	0	0	0	1	0
PF05016	Sberro et al., 2013	TA	ParE toxin of type II toxin-antitoxin system	1	1	1	1	1
PF05534	Sberro et al., 2013	TA	HicB family	1	1	0	1	0
PF05973	Sberro et al., 2013	TA	Phage derived protein Gp49-like	1	1	1	1	1
PF06296	Sberro et al., 2013	TA	RelE toxin of RelE/RelB toxin-antitoxin system	1	0	1	1	1
PF06414	Sberro et al., 2013	TA	Zeta toxin protein domain	1	1	1	1	1
PF07804	Sberro et al., 2013	TA	HipA-like C-terminal domain	1	1	1	1	1
PF07927	Sberro et al., 2013	TA	HicA toxin of bacterial toxin-antitoxin	0	0	0	1	0
PF08845	Sberro et al., 2013	TA	Toxin SymE, type I toxin-antitoxin system	0	0	1	0	1
PF11663	Sberro et al., 2013	TA	Toxin with endonuclease activity	0	0	0	1	0
PF13560	Sberro et al., 2013	TA	helix-turn-helix (HTH) motif capable of binding DNA	0	1	1	1	1
PF13657	Sberro et al., 2013	TA	HipA N-terminal domain	1	1	1	1	1
PF15738	Sberro et al., 2013	TA	Bacterial toxin of type II, YafQ-like	0	1	1	1	0
PF15937	Sberro et al., 2013	TA	prlF antitoxin for toxin YhaV_toxin	0	0	0	1	0

**^*a*^* CRISPR, Clustered Regularly Interspaced Short Palindromic Repeats; RM, Restriction-modification; TA, Toxin-antitoxin; Abi, Abortive Infection. *^*b*^* 0 and 1 means absence and presence of the Pfam, respectively. (1) means presence of the Pfam but in different strains than those analyzed in this work. 1^∗^ means presence of the Pfam but in different strains, which makes the corresponding defense system to be incomplete and, highly probable, non-functional.*

### Gene Gain and Loss

We estimated the rates of protein families/domains gain, loss, and duplication by applying a birth-and-death model implemented in the software package COUNT v9.1106 (Csúrös, 2010). For this, we employed Table 2 as the primary information of the presence/absence for all proteins, and a rooted species-tree (see below for species tree reconstruction). The rate estimations were calculated using the gain-loss-duplication model with the Poisson distribution, and searching for increasing complexity by three discrete categories for the gamma distribution. When used 4 gamma categories we obtained similar results than with 3 categories. For optimization, 100 rounds were executed to reach the convergence criteria with a likelihood threshold of 0.1.

The species tree for evolutionary analysis was estimated under the Bayesian framework using the software BEAST v1.10.4 (Suchard et al., 2018) coupled to BEAGLE library v2.1 which accelerates the process of calculation (Ayres et al., 2012). Initial data was obtained from concatenating sequence proteins using the BPGA v1.3 software (Chaudhari et al., 2016). This strategy produced an aligned sequence of 25.558 amino acids that correspond to the core genome of the fifteen RSSC strains analyzed in this work. The best model selection for protein substitution across sites was estimated using the online tool SMS^7^ (Lefort et al., 2017). The Bayesian phylogenetic inference was set to the strict clock model with a constant growth for the tree prior. The shape (α) parameter of the gamma distribution and the proportion of invariant sites (pInv) were set up to lognormal distribution with initial value and mu (μ) equal to 0.5. The analysis was run for 25 million generations, sampling every 2,000 generations. The convergence of the MCMC chains was assessed by evaluating the Effective Sample Size (*ESS*) of all parameters using Tracer v1.7.1 (Rambaut et al., 2018). We summarized the posterior sample of trees generated by BEAST to produce the maximum clade credibility tree using TreeAnnotator v1.10.4.

### Horizontal Gene Transfer (HGT) Analysis

To search for HGT events in the defense system genes, we employed the software Notung v2.9 (Chen et al., 2000) that reconciles gene trees with the species tree to infer duplication-transfer-loss event models with a parsimony-based optimization criterion for each Pfam (Stolzer et al., 2012). We selected the ‘Prefix of the gene’ option as the species label to reconcile genes and species phylogenetic trees. To detect HGT events, Notung requires rooted trees. For this, we employed the maximum clade credibility tree obtained previously (see above) using BEAST, which corresponds to the species tree. Then, we reconstructed gene trees in BEAST using similar strategies and settings than for species tree. Briefly, protein datasets were created for each Pfam, based on Table 2. Each protein sequence was retrieved from the NCBI’s FTP server^8^ If more than one protein sequence existed for the same bacterial strain and a given Pfam, we used a consensus seed alignment that contained the most conserved sequences. Not enough Druantia homologous proteins were found to construct a robust gene tree, therefore there are no results about HGT in this defense system. The protein datasets were aligned using the MAFFT aligner (Katoh et al., 2019) and then used to reconstruct gene trees in BEAST v1.10.4 with the BEAGLE v3.0.1 library (Ayres et al., 2012), using similar strategy and settings than for species tree. The aligned protein sequences of each Pfam were tested for the best model selection for protein substitution using SMS software (Lefort et al., 2017). The phylogenetic reconstruction was set up to JTT as the model of amino acid substitution that best fit in all datasets with gamma distribution and invariant sites. The alpha and pInv parameters were set up to lognormal with μ = 0.5 – 5.0 and σ = 1.0. MCMC was run for 20 million generations to ensure stationary and convergence of parameters was assessed by calculating the ESS using Tracer v1.7.1. Like above, the maximum clade credibility (MCC) trees were summarized using TreeAnnotator v1.10.4 and visualized with FigTree v1.4.4.

Horizontal gene transfer events inferred by Notung for each defense system were displayed and visualized as a donor-recipient network using Gephi v0.9.2 (Bastian et al., 2009). For this, we created ‘edge tables’ that contained the recipient and donor information of an HGT event. The graph type was set as undirected (i.e., without edge direction) and we used the Force Atlas 2 layout algorithm with scaling = 1000, stronger gravity to make cluster tighter, and overlap prevention.

### Association of Defense Systems and Non-defense Related Genes

We downloaded protein sequences of basal metabolism enzymes, effectors (T3E) and cell-wall-degrading enzymes (CWDE) from protein databank^9^. We confirmed that all basal metabolism enzymes selected in this study are present in all RSSC strains analyzed here using BLASTp. Information about the presence/absence of T3E in the genomes of the strains analyzed in this study was obtained from https://iant.toulouse.inra.fr/T3E. For the presence/absence of CWDE in the RSSC genomes, we used BLASTp and respective protein sequences downloaded from strain GMI1000 as query data. The basal metabolism enzymes, T3E and CWDE protein sequences were useful to find their respective Pfam accessions using the HMMER web server, as explained above. A binary matrix was created of defense systems and basal metabolism enzymes, using Pfam accessions for both groups. A similar approach was performed to develop a matrix for defense systems and T3E and CWDE. Each binary matrix was uploaded in the BAYESTRAITS v3.0.1 software package (Pagel, 1994) together with the phylogenetic tree generated in BEAST v1.10.4 (see above) to test the evolutionary association between the defense systems and basal metabolism enzymes, the defense systems and T3Es or the defense systems and CWDEs. In general, we followed the methodology described in Press et al. (2013). Briefly, we analyzed discrete traits evolution under independent or dependent assumptions. We used the ML approach and repeated the analysis by calling the ML algorithm 100 times, which produces more stable results. To establish whether the dependent or independent model of evolution fits better the data, we employed the likelihood ratio test (LRT). For the LRT, we used the log likelihood of both models of evolution generated by BAYESTRAITS and was computed as 2[Lh(D)-Lh(I)]. A Chi squared test significance that equals 9.49 for a significance level of 0.05 and 4 degrees of freedom (the dependent model has 8 rate parameters and the independent model has 4) was used. The likelihood ratios less than this critical value were considered independent.

### Estimation of Population Recombination and Mutation Rate Parameters

We aligned all available DNA sequences for each Pfam of defense systems (Table 2) using the MAFFT online server^10^ (Katoh et al., 2019). We excluded from the analysis, the Pfams with an insufficient number of sequences (less than 5 sequences) to calculate the recombination and mutation parameters. The recombination rate, rho (ρ) per site and the mutation rate, theta (θ) per site were calculated for each aligned set of sequences using the RDP v.4.97 (Martin et al., 2015) following default settings. The θ calculated is also known as the Watterson’s θ, which estimates the genetic diversity in a population and, at the same time, it is suitable for measuring the mutation rate of a population (Watterson, 1975).

## Results

### Broad Diversity of Defense Systems in RSSC

Throughout evolution, bacteria have developed an ample arsenal of mechanisms to defend themselves from the attack of phages and other mobile elements such as plasmids. RSSC is not the exception. By matching the Pfam and COG entries of well-known defense components in bacteria with the respective entries of RSSC genomes, we confirmed the presence of certain protein families and domains with a potential role in defense. In this way, we identified numerous defense systems in RSSC phylotypes (Table 2). We have focused our search on bacterial systems with greater distribution in all bacteria, but not in systems that are specific to particular groups such as the *Dpd* cluster in *Salmonella enterica* (Thiaville et al., 2016) and particular cases in *Mycobacterium*, *Pseudomonas* and *Gordonia* (Bondy-Denomy et al., 2016; Dedrick et al., 2017; Montgomery et al., 2019). We also did not pay attention to defense systems that are virus-mediated such as *sie* (superinfection exclusion, Broecker and Moelling, 2019).

**FIGURE 1:**
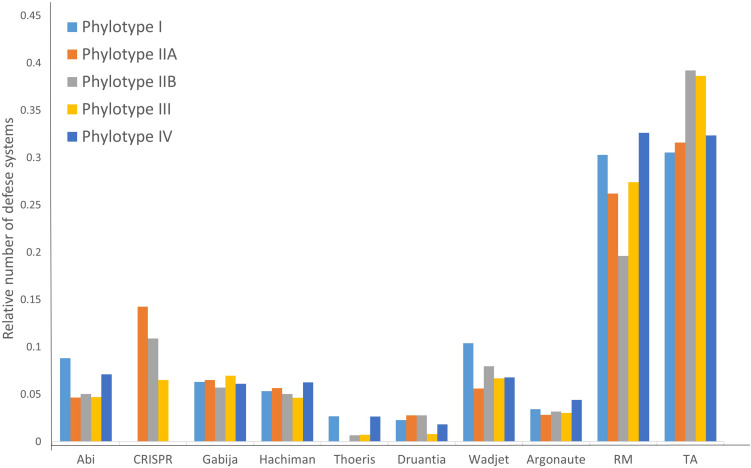
Distribution of the relative abundance of defense systems in the RSSC.

**FIGURE 2:**
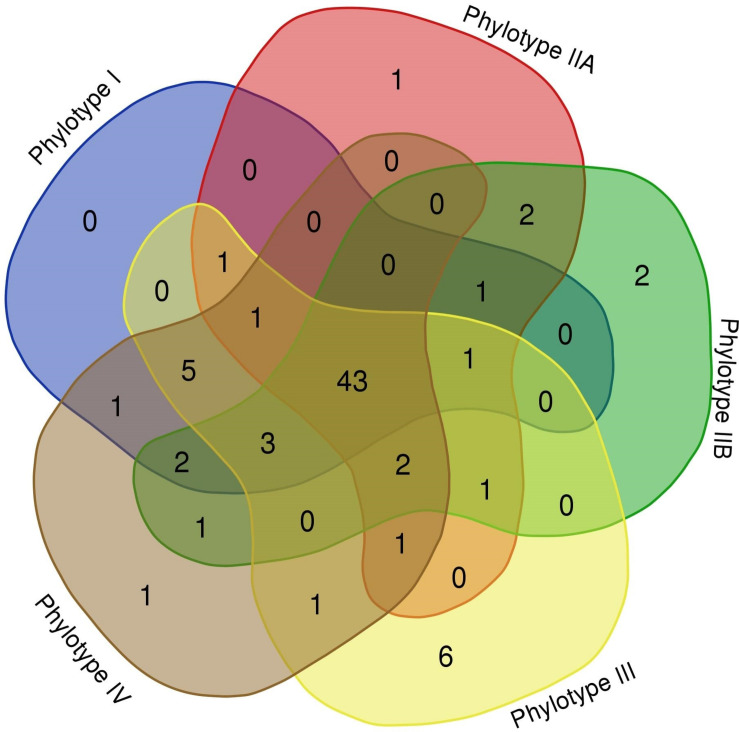
Venn diagram of the RSSC defense system pan-genome. A “flowerplot” of protein families in phylotypes of the RSSC. Numbers correspond to Pfam entries in phylotypes.

As seen in Table 2, different RSSC phylotypes harbor diverse systems. In general, we found nine different systems devoted to defense from phage attack and one against plasmid transformation. Among the antiphage systems, the toxin-antitoxin system comprises the largest group of defense systems (on average 34.5% of the total defense systems in RSSC), followed by restriction-modification (27%) and Wadjet (7.5%, Figure 1). Additionally, a pan-genomic analysis of the fifteen RSSC genomes revealed that the defense systems are composed of a core of 43 protein families (Pfams) and, 10 families are unique among different phylotypes, being the phylotype III the one that harbors more unique protein families (Figure 2). Each defense system in RSSC is described below.

#### Toxin-Antitoxin System (TA)

Twenty-eight family proteins (Pfam accessions) make up modules of two contiguous genes that encode the toxin and its cognate antitoxin, representing the TA system in RSSC. Of these families, five are involved in the abortive infection (Abi) process that protects bacteria from phage infection through an altruistic suicide mode (Table 2). Abi systems work causing the death of the infected cells as a sacrifice to protect the surrounding cells from future predation (Seed, 2015).

#### Restriction-Modification (RM)

The RM system shows eighteen protein families in RSSC. Just like the TA system, all phylotypes harbor RM systems. Two main proteins form this system: a restriction endonuclease, and an enzyme for methylation. Eventually, a third member that adds specificity participates in this system. Likewise, a second methylase or methyltransferase may accompany the gene cluster or two methylases/methyltransferases reside close in the genome without the endonuclease. This diversity of proteins allows the organization of this system in four main types as is found in most of the bacterial species. We found that all four types of RM systems are present in different strains of RSSC, although the most abundant is Type II that is composed of the methyltransferase and the endonuclease encoded as two separate and independent proteins (Supplementary Table 2).

#### Clustered Regularly Interspaced Short Palindromic Repeats (CRISPR)

The complete set of protein families that correspond to the CRISPR associated proteins (Cas) is present in the strains studied in this work that belongs to phylotypes IIA, IIB and III (strains CFBP2957, CIP120, IBSBF1503, Po82, and CFBP3059). A genomic analysis of the palindromic repeats and spacer content indicates that these strains have the Type I-E CRISPR-Cas system (Supplementary Table 3). This system utilizes numerous Cas proteins for the recognition and cleavage of targeted nucleic acids, therefore the genome of the RSSC strains contains the following Cas proteins: Cas1_0_IE, Cas2_0_IE, Cas3_0_IE, Cas5_0_IE, Cas6_0_IE, Cas7_0_IE, Cse1_0_IE, and Cse2_0_IE. Besides the mentioned strains, other strains from phylotypes I and IV not included in Table 2 show the presence of CRISPR-Cas system as da Silva, Xavier et al. (2019) reported. This result indicates that the CRISPR-Cas system is present in all phylotypes although it is not widely distributed across strains.

#### Gabija

This system is composed of two main components, a DNA helicase (UvrD/REP type) with AAA domain (GajB) and an ATP dependent endonuclease with ATPase domain (GajA). The *gajAB* genes are arranged in an operon and they are present in two copies in the strain PSI07 and one copy in CIP120, and KACC10722. The other strains (HA4-1, IBSBF1503, CMR15, CFBP3059, and UW386) that contain the genes of this system are dispersed in the genome (highlighted with an asterisk in Table 2); therefore, it is very likely that they do not fulfill a biological function as a defense system.

#### Hachiman

This system is also composed of two genes *hamAB* that encodes a protein with unknown function (HamA, Pfam accession: 08878) and a helicase (HamB). Only one strain (KACC10722) from phylotype IV shows this system complete and both genes are organized in an operon.

#### Thoeris

This system is characterized by gene, *thsB*, encoding a protein with a TIR domain. This gene is typically preceded by *thsA*, a gene that encodes a protein containing a NAD-binding domain that is commonly annotated as SIR2-like domain. In RSSC, this system is found in phylotype I (strain GMI1000) and phylotype IIB (strain IBSBF1503). No other strain of another phylotype –from our set of strains- has both genes, but the system is incomplete in strains PSI07, KACC10722, CMR15 (which contain only *thsA*) and T98, HA4-1, FQY_4 (which contains only *thsB*). Since both Thoeris genes are essential for the normal functioning, these strains most likely have the system inactive.

#### Druantia

This system comprises five genes in RSSC: *druA*, encodes a large protein with a domain with unknown function (DUF4338), *druBCD* with unknown function and no protein family designation and *druE* that encodes also a large protein with DUF 1998 domain as well as a helicase signature and ATP-binding motif. This arrangement of genes corresponds to the so-called Type I Druantia system and it is not found in any of the strains studied in this work but in other strains of RSSC. Certainly, strain UW163 (Phylotype IIB) harbors the complete set of genes which indicates that this system would be fully functional in this strain. Other strains contain the incomplete system (T82, Grenada 9-1, T101, UW181, SL3822, SL2312, P822, SL3022, UA-1611, BBAC-C1, UW386, and T12).

#### Argonaute

We found the ‘short Argonaute’ type system in RSSC. Two contiguous genes that encode a nuclease with PIWI (acronym of the *P*-element Induced Wimpy Testis) domain and a protein of SIR2 family characterize this system. No strains of our set of strains contain this system, however, it is present in strains: OE1-1, EP1, PSS1308, and VT0801 (phylotype I). Incomplete versions of this Argonaute system are shown in different strains (highlighted with an asterisk in Table 2) but we note that they probably have no biological function as a defense system.

#### Wadjet

This is the only defense system dedicated to reducing the incidence of plasmid transformation in bacteria. It consists of four genes, *jetABCD*, with unknown function except for *jetC* that encodes a protein with an *N*-terminal domain that is found in the system that performs structural maintenance of chromosomes and harbors an ATP-binding motif. The Wadjet system is broadly disseminated in RSSC, being possible to find it in all phylotypes: I (GMI1000, FQY_4, YC45, and SD54); IIA (K60); IIB (Po82, P673, UW 163, IBSBF1503); III (CMR15); IV (PSI07, KACC10722).

### Gain and Loss of Genes During the Evolution of RSSC Defense Systems

To unravel the evolutionary process of the defense systems in RSSC, we focused to study gene content evolution. For this, we used the COUNT software that applies maximum likelihood and the birth-death model that can take into account the effects of different evolutionary mechanisms of gene gain and loss when analyzing gene family data. It is feasible to model the evolution of protein families because losses and gains seem to occur independently between the members of multigene families (Nei and Rooney, 2005). Therefore, the most general process of the gene family evolution is gain (most common through HGT), loss and duplication. We calculated the rate of gene family gain, loss, and duplication for defense systems as well as for all genes encoding proteins in the genomes of RSSC.

Results indicate that, contrary to what is observed in other bacterial systems in which gene loss has dominated the evolution of defense systems (Puigbò et al., 2017), in RSSC, defense genes show a propensity for gene gain. In fact, the average rate of gene gain versus loss is 5-fold higher and the duplication rate being approximately 1.9-fold higher for all strains analyzed in this work (Table 3). Our results indicate that, although in many defense systems, several genes have been lost in terminal edges as is the case of Gabija, Abi, Thoeris, other systems have experienced an early surge in gene content, which gave rise to a wide range of orthologous genes in the current strains. This is the case of CRISPR that was probably acquired early when RSSC was divided into the phylotypes that we observe today. This fact is evidenced by observing a gene gain occurrence close to the root of the tree (Supplementary Figure 1). When comparing the rate of gene gain and loss of defense systems with all genes in the RSSC genomes, we observe an inclination toward a net loss of all gene families in the RSSC genomes (Table 3).

**TABLE 3:** Genome dynamic events in defense systems in all studied strains.

**Strains**	**Evolutionary rates**					
	**Defense systems**			**Whole genome**		
	**Loss**	**Duplication**	**Gain**	**Loss**	**Duplication**	**Gain**
PSI07	0.41	0.8	2.3	0.58	0.53	0.24
T98	0.27	0.52	1.5	0.18	0.17	0.07
KACC10722	0.21	0.41	1.2	1.0	0.9	0.4
GMI1000	0.11	0.21	0.61	0.09	0.08	0.04
HA4-1	0.14	0.27	0.77	0.12	0.11	0.05
FQY_4	0.08	0.16	0.45	0.07	0.06	0.03
CMR15	0.23	0.44	1.3	0.11	0.1	0.05
CFBP3059	0.14	0.28	0.80	0.1	0.09	0.04
UW386	0.35	0.68	1.9	0.4	0.36	0.16
RS489	0.25	0.48	1.4	0.47	0.43	0.19
CIP120	0.16	0.31	0.9	0.09	0.09	0.04
CFBP2957	0.07	0.14	0.41	0.34	0.31	0.14
UW551	0.07	0.13	0.39	0.07	0.07	0.03
IBSBF1503	0.17	0.33	1.0	0.4	0.36	0.16
Po82	0.01	0.02	0.06	0.04	0.04	0.02
Average	0.18	0.35	1.0	0.27	0.25	0.11

### Extensive Horizontal Transfer of RSSC Defense System Genes

Horizontal gene transfer is probably the main mechanism through which defense genes have been gained in RSSC genomes. We tested to confirm and measure the extent of HGT in the defense systems using a well-known approach aimed at reconciling the gene tree with the reference species tree. Donor-recipient networks summarizing HGT events are shown in Supplementary Figure 2 for most of the defense systems analyzed in this work. Only one optimal solution (tree) was considered for each Pfam and all Pfams for a defense system is briefed in Supplementary Figure 2. Multiple optimal solutions occur when transfer events are a dominant process, according to Notung. Donor and recipient strains/clades vary from one optimal solution to another. It should also be pointed out that we were unable to track HGT events for all Pfams mainly due to the low number of sequences available for some Pfams or the impossibility of Notung to calculate a temporally feasible solution of possible HGT events. This happens when a transfer (HGT event) occurs and both, recipient and donor had to co-exist in the same time interval (Stolzer et al., 2015).

As it is possible to observe in Supplementary Figure 2, the HGT events were profuse between strains of RSSC. Pfams of the Argonaute, Gabija, Hachiman, RM, TA-Abi, and Wadjet have undergone several transfer and loss events across their evolutionary history. Conversely, few events of HGT were detected in CRISPR-Cas system and Thoeris, which have been restricted mainly to the tips of the trees (reconciled trees not shown) and only present in a few strains. In the case of CRISPR-Cas proteins, they are highly conserved; this may be due, precisely, to the low horizontal transfer rate observed in this system.

### Association of Defense Systems Evolution With Essential and Pathogenicity Functions

The results of the previous analysis bring to light that the genes encoding the defense systems may follow a different and independent regime of evolution than the rest of the genes in the genomes. To confirm this presumption, we set out to correlate defense systems evolution with other cellular systems devoted to essential functions or pathogenicity. We selected a group of genes encoding enzymes of the basal metabolism to examine whether there is an evolutionary association between defense systems and essential (housekeeping) enzymes. For pathogenicity, we chosen the proteins secreted through the type III secretion system, better known as the ‘effectors’ (called T3E hereafter) and cell-wall-degrading enzymes (CWDE). We used a novel method that performs analyses of trait evolution among groups, genes or systems for which phylogeny is available. This method uses a continuous-time Markov process to evaluate different models of evolution and estimates each of these rate parameters by maximum-likelihood. Then, the best evolutionary model for the particular data under analysis is selected by computing the likelihood ratio test (LRT) using likelihood scores. To analyze RSSC data, we created a binary matrix to compare two groups of genes: defense system vs basal metabolism (housekeeping enzymes) or defense system vs pathogenicity (T3E or CWDE) within a phylogenetic tree and determined if changes in the two groups have evolved independently or dependently. In this way, we estimated the extent of association or participation of defense systems in basal metabolism or pathogenicity. Results indicate that all pairwise comparisons between systems generate values of the LRT below the critical value demonstrating that there is no significant association between evolution of defense systems and basal metabolism and neither between defense systems and pathogenicity (see Supplementary Table 4). This implies that defense systems in RSSC must have evolved independently from other systems (at least, independently from basal metabolism and pathogenicity determinants).

### Higher Molecular Evolution Rate in Defense System Genes Than in Genomic Regions

The analysis performed above suggests that defense systems follow an independent evolution, unlinked to other cellular systems (i.e., basal metabolism or pathogenicity). This implies that a similar discrepancy between the evolutionary rate of defense systems and other systems must be observed. Therefore, we wonder what the rate of molecular evolution of defense systems is compared to the general rate of RSSC genomes. For this estimation, we used two different estimators, recombination rate (ρ) and mutation rate (θ) as proxies of the rate of molecular evolution. Both estimators provide population-scaled data so they are useful for getting an idea about the molecular evolution rate of the defense systems in the RSSC population. We calculated ρ and, θ for each aligned sequence corresponding to the Pfams of the defense systems. However, some defense systems are rare in RSSC, namely, they are present in only a few strains (such as Argonaute), therefore, it was not possible to include in the analysis the systems that lacked the minimum critical number of sequences to perform the calculations. The average values of ρ and θ calculated for 48 Pfams are 0.01 and 0.05 respectively (Supplementary Table 5). These values are 1.85 and 4.54 times higher than the respective values calculated for genomic sequences according to Castillo and Agathos, 2019. This result indicates that the relative contribution of recombination and mutation to the evolution of defense systems is higher than the rest of the genome in RSSC.

## Discussion

We set out to describe the diversity of defense systems that are present in the phylotypes of RSSC. We found nine protein families of different systems devoted to defense from phage attack and one linked to reducing plasmid transformation. The density of defense systems in RSSC genomes varies over broad range: some defense systems are widely extended in all phylotypes (i.e., RM, TA) whereas others are restricted to few strains in one or two phylotypes (i.e., Argonaute, Gabija). Although the number of defense systems in RSSC is significant, we do not rule out that computer and experimental means might identify other cryptic systems.

In this work, we did not perform *in vivo* experiments to determine the antiphage or anti-plasmid efficacy of the systems; however, we based our analysis on the results of different colleagues who experimentally validated all of the protein families for the defense capacity in many other bacteria and archaea groups. Besides, the defense systems are widely distributed in bacteria so it is not rare to find them in RSSC. The RM and TA systems are thought to be ubiquitous in bacteria (Makarova et al., 2013) but, other systems, although present in a smaller proportion, are no less important since they were found in many bacterial genomes (i.e., Gabija and Wadget are present in 8.5% and 5.6% of bacterial genomes analyzed, respectively, Doron et al., 2018).

The mechanism of action to abolish phage attack is known for some systems (e.g., RM, TA), but is not yet clear for other systems such as Argonaute (Swarts et al., 2014), and the recently discovered ones (e.g., Thoeris, Druantia, or Wadjet) (Doron et al., 2018). Some defense systems in RSSC are particularly interesting to describe. The Argonaute system is found in the three main domains of life (Bacteria, Archaea, and Eukaryota) and is broadly distributed in both archaea (∼30% of all sequenced genomes) and bacteria (∼10% of genomes). It has several possible cellular functions: it can participate in the regulation of the transcriptional expression of host genes, it might act as a suicide system similar to abortive infection systems that kill a bacterial host under stress conditions and it works as defense against foreign genetic elements such as transposons, phages, and plasmids (Lisitskaya et al., 2018). In Betaproteobacteria (the class where RSSC is taxonomically located) most Argonaute proteins are short-type with only MID and PIWI domains (Ryazansky et al., 2018), and likewise, this type has been found in RSSC. Although this system is poorly distributed in RSSC phylotypes, it seems to be complete in a few strains of phylotype I, although we do not rule out that it might be present in strains of other phylotypes.

The CRISPR-Cas system in RSSC was first described by da Silva, Xavier et al. (2019). They found this system in 31% of RSSC genomes present in public databases. We found in 5 out of 15 strains, which corresponds to approximately the same percentage (33%). Similarly, our results of gene content analysis and HGT indicate that CRISPR-Cas system is ancient and that would have been present in RSSC before the split in phylotypes, agreeing with da Silva, Xavier et al. (2019) results that point out the early acquisition of this system by a common ancestor before *Ralstonia* species segregation.

Thoeris system works to reduce or control the entry of plasmids into the bacterial cell (Doron et al., 2018). We found this system in a few strains of our set of strains analyzed here; however, we do not rule out that other strains can also harbor this system. Conversely, it is well-known the competency of RSSC to natural transformation, so that many strains can exchange DNA fragments up to 90 Kb (Coupat et al., 2008). How can we accommodate these two seemingly contradictory functions? Most likely, a dynamic equilibrium of both functions occurs in parallel inside the cell, which guarantees the genetic diversification without the burden of taking useless DNA fragments.

We have used different methods to study the evolutionary dynamics of defense systems in RSSC, which offer concordant and complementary results. All the evidence collected in this work on the evolution of defense systems in RSSC indicates that they have been principally gained as opposed to the rest of genes present on the RSSC genomes that are preferably lost (Table 3). This result is consistent with that reported by Lefeuvre et al. (2013), which indicates that the gene function is the key factor in the gene gain and loss dynamics in RSSC. We have also found some traces of gene duplication in a few defense systems mostly at the base of trees or ancestral nodes. Thereby, gene gain and duplication are the main forces that have driven the expansion of the defense gene content in RSSC. Contrary, it has been demonstrated that the dominant mode of evolution of defense systems in other bacterial groups is gene loss (Puigbò et al., 2017), but that does not seem to be the case in RSSC. Undoubtedly, the main mechanism of gene gain is HGT, which has played a significant role in shaping defense systems in RSSC. Results of tree reconciliation to detect HGT events (Supplementary Figure 2) show a profuse transference of genes between RSSC strains and phylotypes. This abundant transference of genes in RSSC is not surprising since other studies reported multiple DNA acquisitions along the genome through HGT events (Guidot et al., 2009).

We tested the evolutionary association of defense systems with other non-defense systems such as essential (housekeeping) and pathogenicity (T3E or the CWDE) functions. Results provided by the BayesTraits program suggest that the defense systems of RSSC follow an independent evolutionary pattern than other cellular systems. In other words, the evolution of these systems is not correlated among them, suggesting that defense systems follow an independent evolutionary regime than the other functions. Maybe this is because the defense systems are subject to different selective pressures, which forces different evolutionary rates than the rest of the cellular functions. Indeed, we found different evolutionary rates in the defense systems than the rest of the genome, when we calculated the rates of recombination and mutation (Supplementary Table 4).

The abundance and diversity of defense systems in RSSC implies that they play an important role as a major line of innate defense against a great diversity of phages (see Table 1) that reside in the different natural environments where RSSC strains live. The continuous process of defense and counter-defense mechanisms must constantly evolve to maintain the fitness of both interacting partners. This coevolutionary process generates an enormous phage diversity, which in turn have triggered an adaptive race for increasing resistance in RSSC.

Although much work remains to be done, especially at the experimental level, this study opens the door for further research focused on understanding the dynamic world of RSSC and its parasites. Our study is also useful for designing better phage therapy strategies. An important problem in phage therapy is that bacteria may evolve resistance to phages, thus making the use of phages fruitless. The knowledge of the defense systems present in particular strains of RSSC can help select more carefully the appropriate phages to avoid possible resistance. Likewise, studies on the evolutionary dynamics of RSSC-phage interaction could provide useful information about evolutionary parameters such as the fitness cost to maintain resistance to phage types. Alternatively, it would be possible to design experimental evolution assays (as is the case of *Pseudomonas syringae* and four related phages, Koskella et al., 2012) to increase the spread, infectivity, and persistence of phages in natural environments where RSSC survives.

## Data Availability Statement

The genomic data analyzed in this study can be found in the NCBI database, see Supplementary Table 1 (in Supplementary Data Sheet 1) for details.

## Author Contributions

HS-M and SM performed the phylogenetic and HGT analyses. KS analyzed evolutionary association. JC conceived and designed the study, analyzed the genomic data, calculated evolutionary rates and wrote the manuscript. All co-authors contributed to the manuscript revision, read, and approved the submitted version.

## Conflict of Interest

The authors declare that the research was conducted in the absence of any commercial or financial relationships that could be construed as a potential conflict of interest.

## Footnotes

1 https://www.ncbi.nlm.nih.gov/genome/microbes/

2 https://www.ebi.ac.uk/Tools/hmmer/search/hmmscan

3 https://www.ncbi.nlm.nih.gov/Structure/bwrpsb/bwrpsb.cgi

4 https://crisprcas.i2bc.paris-saclay.fr/CrisprCasFinder/Index

5 http://crispi.genouest.org/

6 http://rebase.neb.com

7 http://www.atgc-montpellier.fr/phyml/

8 https://www.ncbi.nlm.nih.gov/nuccore

9 https://www.ncbi.nlm.nih.gov/protein/

10 https://mafft.cbrc.jp/alignment/server/
