# Supplementary material for: Diversity and Evolutionary Dynamics of Antiphage Defense Systems in Ralstonia solanacearum Species Complex
Source: Front Microbiol. 2020 May 20;11:961. doi: 10.3389/fmicb.2020.00961 (PMC7251935; doi:10.3389/fmicb.2020.00961)

Supplementary Material

# Supplementary Tables

**Supplementary** **Table 1.** Strains of RSSC used in this work.

| Strain name | Phylotype/sequevar | Genomic sequence accession |
| --- | --- | --- |
| GMI1000 | I-18 | NC_003295.1/NC_003296.1 |
| FQY_4 | I-14 | NC_020799.1/NC_021745.1 |
| HA4-1 | I-54 | NZ_CP022481.1/NZ_CP022482.1 |
| CFBP2957 | IIA-36 | FP885897.1 |
| RS489 | IIA-50 | NZ_CP021766.1/NZ_CP021767.1 |
| CIP120 | IIA-38 | JXAY01 |
| Po82 | IIB-4 | NC_017574.1/NC_017575.1 |
| UW551 | IIB-1 | AAKL01 |
| IBSBF1503 | IIB-4NPB | NZ_CP012943.1/NZ_CP012944.1 |
| CMR15 | III-29 | NC_017559.1/NC_017558.1 |
| CFBP3059 | III-23 | JXBA01 |
| UW386 | III | NZ_CP039339.1/NZ_CP039340.1 |
| T98 | IV | NZ_CP022759.1/NZ_CP022760.1 |
| PSI07 | IV-10 | NC_014311.1/NC_014310.1 |
| KACC10722 | IV | NZ_CP014702.1/NZ_CP014703.1 |

**Supplementary** **Table 2.** Distribution of Restriction-modification systems in the 15 genomes of RSSC analyzed here.

| **Strain/**  **gene locus tag** | **Type** | **RM component** |
| --- | --- | --- |
| **GMI1000** |  |  |
| RSc3394 | I | Restriction modification enzyme R |
| RSc3396 | I | Restriction enzyme M protein |
| RSc0844 | II | DNA modification methylase protein |
| RSc0845 | II | DNA modification methylase protein |
| RSc3438 | II | Site-specific DNA methylase protein |
| RSc3439 | II | VSR patch repair protein (DNA mismatch) |
| RSp0570 | II | DNA methylase N6 adenine CS, SAM-dependent Mtases |
| RSc1982 | II | DNA-methyltransferase (DNA-modification) |
| RSc0869 | II | Bacteriophage cytosine-specific methyltransferase protein |
| **FQY_4** |  |  |
| F504_3443 | I | Methyltransferase |
| F504_3445 | I | Type I restriction-modification system |
| F504_1929 | II | Adenine-specific methyltransferase |
| F504_3074 | II | DNA methylase |
| F504_3240 | II | Adenine-specific methyltransferase |
| F504_3241 | II | DNA modification methylase |
| F504_3478 | II | DNA-cytosine methyltransferase |
| F504_4033 | II | Adenine-specific methyltransferase |
| F504_4032 | II | Methyltransferase |
| **HA4-1** |  |  |
| CFM90_16320 | I | SAM-dependent DNA methyltransferase |
| CFM90_16330 | I | Restriction endonuclease subunit R |
| CFM90_05085 | II | DNA-methyltransferase |
| CFM90_09275 | II | DNA cytosine methyltransferase |
| CFM90_10885 | II | Methyltransferase |
| CFM90_11015 | II | DNA methylase N-4 |
| CFM90_11020 | II | DNA modification methylase |
| CFM90_11620 | II | Methyltransferase |
| CFM90_15070 | II | DNA methylase |
| CFM90_16070 | II | Very short patch repair endonuclease |
| CFM90_16075 | II | DNA (cytosine-5-)-methyltransferase |
| CFM90_17800 | II | DNA methylase N-4 |
| CFM90_19905 | II | DNA methylase N-4 |
| CFM90_16220 | IV | Endonuclease |
| **CFBP2957** |  |  |
| RCFBP_10068 | I | Type I restriction enzyme (hsdM) |
| RCFBP_10070 | I | Restriction endonuclease, type I, R |
| RCFBP_10010 | II | Cytosine-specific methyltransferase |
| RCFBP_11404 | II | Site-specific DNA-methyltransferase (adenine-specific) |
| RCFBP_20378 | II | DNA (cytosine-5-)-methyltransferase |
| **RS 489** |  |  |
| CDC59_00455 | I | SAM-dependent DNA methyltransferase |
| CDC59_00460 | I | Hypothetical protein, possible restriction enzyme |
| CDC59_05550 | II | Site-specific DNA-methyltransferase |
| CDC59_05565 | II | Hypothetical protein, possible restriction enzyme |
| CDC59_05570 | II | Site-specific DNA-methyltransferase |
| CDC59_05600 | II | Class I SAM-dependent DNA methyltransferase |
| CDC59_11390 | II | Site-specific DNA-methyltransferase |
| CDC59_23465 | II | DNA modification methylase |
| CDC59_23470 | II | DNA methylase N-4 |
| **CIP120** |  |  |
| RSP795_01845 | I | DNA methyltransferase |
| RSP795_01850 | I | Restriction endonuclease subunit R |
| RSP795_16065 | I | DNA methyltransferase |
| RSP795_16075 | I | Restriction endonuclease subunit R |
| RSP795_06335 | II | Methyltransferase |
| RSP795_21850 | II | Methyltransferase |
| RSP795_22230 | II | Methyltransferase |
| RSP795_24595 | II | Methyltransferase |
| RSP795_24720 | II | Methyltransferase |
| RSP795_22225 | II | DNA methylase N-4 |
| RSP795_07305 | II | HNH endonuclease |
| RSP795_10765 | II | UvrABC system protein B (Protein UvrB) |
| RSP795_03295 | II | DNA methyltransferase |
| RSP795_15955 | II | Endonuclease |
| RSP795_15915 | II | Type I site-specific restriction-modification system, R subunit. |
| RSP795_15930 | II | Restriction endonuclease subunit M |
| **Po82** |  |  |
| RSPO_c00042 | I | HsdM typeIrestriction enzyme M protein |
| RSPO_c00046 | I | Restriction modification enzyme R subunit |
| RSPO_c00236 | II | Site-specific DNA-methyltransferase |
| RSPO_c00237 | II | Putative DNA-methyltransferase |
| RSPO_c01458 | II | DNA-methyltransferase (DNA-modification) |
| RSPO_c01535 | II | DNA methylase |
| RSPO_c02518 | II | DNA modification methylase protein |
| RSPO_c02519 | II | DNA modification methylase protein |
| RSPO_c02810 | II | Helicase domain protein |
| **UW551** |  |  |
| B7R79_15960 | I | DNA methyltransferase |
| B7R79_15950 | I | Restriction endonuclease subunit R |
| B7R79_10815 | II | SAM-dependent methyltransferase |
| B7R79_10845 | II | DNA methyltransferase |
| B7R79_10850 | II | Restriction endonuclease |
| B7R79_10870 | II | Site-specific DNA-methyltransferase |
| B7R79_05015 | II | Site-specific DNA-methyltransferase |
| B7R79_16060 | IV | Endonuclease |
| **IBSBF1503** |  |  |
| RALBFv3_09185 | I | DNA methyltransferase |
| RALBFv3_09175 | I | Restriction endonuclease subunit R |
| RALBFv3_02580 | II | DNA methylase N-4 |
| RALBFv3_08175/80 | II | Methyltransferase |
| RALBFv3_08290 | II | DNA methylase N-4 |
| RALBFv3_08295 | II | DNA methylase N-4 |
| RALBFv3_09360 | II | Modification methylase PaeR7I |
| ALBFv3_13440 | II | DNA methylase N-4 |
| RALBFv3_13445 | II | DNA methylase N-4 |
| **CMR15** |  |  |
| CMR15_10019 | I | Type I site-specific deoxyribonuclease |
| CMR15_10020 | I | Fragment of restriction endonuclease |
| CMR15_10021 | I | Fragment of restriction endonuclease |
| CMR15_10022 | I | Type I restriction-modification system |
| CMR15_10073 | I | Type I restriction enzyme (hsdM) |
| CMR15_10075 | I | Restriction endonuclease, type I, R |
| CMR15_30585 | I | Putative type I restriction-modification system |
| CMR15_30586 | I | Type I restriction-modification |
| CMR15_30587 | I | Type I restriction-modification methylase M |
| CMR15_10477 | II | Protein of unknown function, possible restriction enzyme |
| CMR15_10478 | II | Cytosine-specific methyltransferase |
| CMR15_11393 | II | Site-specific DNA-methyltransferase (adenine-specific) |
| CMR15_20262 | II | Site-specific DNA-methyltransferase |
| CMR15_30043 | II | DNA modification methylase protein |
| CMR15_30044 | II | Site-specific DNA-methyltransferase |
| CMR15_mp10533 | II | Prophage LambdaMc01, DNA methyltransferase (fragment) |
| CMR15_p0021 | II | DNA-cytosine methyltransferase |
| CMR15_p0022 | II | Restriction enzyme |
| CMR15_p0023 | II | DNA mismatch endonuclease of very short patch |
| **CFBP3059** |  |  |
| RSP799_00130 | I | DNA methyltransferase subunit M |
| RSP799_00140 | I | Restriction endonuclease subunit R |
| RSP799_00130 | I | DNA methyltransferase subunit M |
| RSP799_16100 | II | Cytosine methyltransferase (DNA-cytosine methyltransferase) |
| RSP799_06460 | II | Methyltransferase |
| RSP799_11835 | II | DNA methylase N-4 |
| RSP799_11840 | II | Methyltransferase |
| RSP799_18350 | II | Methyltransferase |
| RSP799_03075 | II | DNA methyltransferase (Putative methyltransferase) |
| RSP799_00005 | II | Endonuclease (Putative endonuclease) |
| RSP799_23970 | II | Methyltransferase |
| RSP799_23965 | II | Methyltransferase |
| **UW386** |  |  |
| E7Z57_00845 | I | Class I SAM-dependent methyltransferase |
| E7Z57_02495 | I | SAM-dependent methyltransferase |
| E7Z57_03245 | I | Class I SAM-dependent methyltransferase |
| E7Z57_05780 | I | Class I SAM-dependent RNA methyltransferase |
| E7Z57_07620 | I | Class I SAM-dependent methyltransferase |
| E7Z57_09365 | I | Class I SAM-dependent methyltransferase |
| E7Z57_12265 | I | Class I SAM-dependent methyltransferase |
| E7Z57_13175 | I | SAM-dependent methyltransferase |
| E7Z57_13185 | I | Class I SAM-dependent methyltransferase |
| E7Z57_01255 | II | Site-specific DNA-methyltransferase |
| E7Z57_01260 | II | Site-specific DNA-methyltransferase |
| E7Z57_04485 | II | Methyltransferase domain-containing protein |
| E7Z57_05800 | II | Methyltransferase domain-containing protein |
| E7Z57_06325 | II | Methylated-DNA-cysteine S-methyltransferase |
| E7Z57_06435 | II | Methylated-DNA-cysteine S-methyltransferase |
| E7Z57_06630 | II | Methylated-DNA-cysteine S-methyltransferase |
| E7Z57_07325 | II | Methyltransferase domain-containing protein |
| E7Z57_10845 | II | Methyltransferase domain-containing protein |
| E7Z57_11565 | II | DNA (cytosine-5-)-methyltransferase |
| E7Z57_11560 | II | DNA mismatch endonuclease Vsr |
| E7Z57_12635 | II | Site-specific DNA-methyltransferase |
| E7Z57_13375 | II | DNA cytosine methyltransferase |
| E7Z57_13485 | II | Site-specific DNA-methyltransferase |
| E7Z57_13490 | II | Site-specific DNA-methyltransferase |
| E7Z57_16225 | II | Methyltransferase domain-containing protein |
| E7Z57_16740 | II | Methyltransferase domain-containing protein |
| E7Z57_13630 | II | Restriction endonuclease |
| E7Z57_14315 | II | Restriction endonuclease subunit R |
| E7Z57_14325 | II | N-6 DNA methylase |
| E7Z57_02300 | II | Endonuclease III |
| E7Z57_03890 | II | Endonuclease/exonuclease/phosphatase family |
| E7Z57_11660 | II | Endonuclease |
| E7Z57_14445 | II | Endonuclease |
| E7Z57_16775 | II | Endonuclease/exonuclease/phosphatase family |
| **T98** |  |  |
| CJO77_00210 | I | DNA methyltransferase |
| CJO77_00220 | I | Restriction endonuclease subunit R |
| CJO77_12670 | II | Type I restriction endonuclease subunit M |
| CJO77_12685 | II | Restriction endonuclease subunit R |
| CJO77_23980 | II | Possible methyltransferase |
| CJO77_00050 | II | Very short patch repair endonuclease |
| CJO77_00055 | II | DNA cytosine methyltransferase |
| CJO77_09085 | III | Restriction endonuclease subunit R |
| CJO77_09090 | III | Site-specific DNA-methyltransferase |
| **PSI07** |  |  |
| RPSI07_0044 | I | Type I restriction enzyme (hsdM) |
| RPSI07_0046 | I | Restriction endonuclease, type I, R |
| RPSI07_mp1589 | II | Fragment of site-specific DNA-methyltransferase |
| RPSI07_0007 | II | Possible restriction enzyme |
| RPSI07_0011 | II | T/G mismatch-specific endonuclease |
| RPSI07_0012 | II | DNA (cytosine-5-)-methyltransferase |
| RPSI07_0227 | II | Site-specific DNA-methyltransferase |
| RPSI07_0228 | II | DNA modification methylase protein |
| RPSI07_1447 | II | Site-specific DNA-methyltransferase |
| RPSI07_2502 | II | Possible methyltransferase |
| **KACC 10722** |  |  |
| LBM2029_00190 | I | DNA methyltransferase |
| LBM2029_00200 | I | Restriction endonuclease subunit R |
| LBM2029_05295 | I | SAM-dependent methyltransferase |
| LBM2029_01010 | II | DNA methylase N-4 |
| LBM2029_01015 | II | DNA methylase N-4 |
| LBM2029_01125 | II | Methyltransferase |
| LBM2029_05210 | II | Restriction enzyme |
| LBM2029_06830 | II | DNA methylase N-4 |
| LBM2029_07740 | II | DNA methylase N-4 |
| LBM2029_00100 | II | Endonuclease |
| LBM2029_16885 | IV | DNA topoisomerase |

**Supplementary** **Table 3.** Distribution of CRISPR-Cas systems in RSSC: CRISPR repeat and spacer content for the strains analyzed in this work.

| **Phylotype/**  **strain** | **CRISPR start** | **CRISPR length** | **Repeat length** | **Consensus repeat** | **Number of CRISPR with same repeat** | **Conservation repeats (% identity)** | **Number of spacers** | |
| --- | --- | --- | --- | --- | --- | --- | --- | --- |
| Phylotype IIA |  | | | | | | |  |
| CFBP2957 | 868741 | 89 | 29 | CGAGCGCCTCCCCGCGCGTGGGCACCACA | 1 | 100 | 1 | |
|  | 1094183 | 332 | 29 | GTGTGTTCCCCGCGTGGGCGAGGATGAGG | 1 | 89.65 | 5 | |
|  | 1104011 | 3567 | 29 | GTGTTCCCCGCGCCTGCGGGGATGAACCG | 4 | 51.72 | 58 | |
| CIP120 | 68371 | 29 | 29 | GTGTTCCCCGCGTGGGCGGGGATGAACCG | 1 | 92.79 | 23 | |
|  | 79240 | 29 | 36 | GTGTTCCCCGCGCCTGCGGGGATGAACCG | 1 | 88.16 | 22 | |
| Phylotype IIB |  | | | | | | |  |
| IBSBF1503 | 955777 | 3630 | 29 | CGGTTCATCCCCGCAGGCGCGGGGAACAC | 1 | 62.06 | 59 | |
|  | 968334 | 2894 | 29 | CGGTTCATCCCCGCCCACGCGGGGAACAC | 1 | 51.72 | 47 | |
| Po82 | 1127636 | 881 | 29 | GTGTTCCCCGCGTGGGCGGGGATGAACCG | 2 | 79.54 | 14 | |
|  | 1137444 | 1005 | 29 | GTGTTCCCCGCGCCTGCGGGGATGAACCG | 4 | 85.88 | 16 | |
| Phylotype III |  | | | | | | |  |
| CFBP3059 | 232 | 24 | 24 | CGGCGGCGACGGCAACGACACCTT | 1 | 49.58 | 2 | |
|  | 628 | 24 | 24 | CGGCGGCGACGGCAACGACACCTT | 1 | 41.03 | 7 | |
|  | 10418 | 29 | 24 | GTGTTCCCCGCGTGGGCGGGGATGAACCG | 1 | 80.81 | 14 | |
|  | 20682 | 29 | 29 | GTGTTCCCCGCGCATGCGGGGATGAACCG | 1 | 99.05 | 20 | |

**Supplementary** **Table 4.** Results of pairwise comparisons to detect evolutionary associations between of defense systems and housekeeping, T3E or CWDE.

Please see attached Excel file: Supplementary Table 4 in data sheet 2_RSSC Defense systems_Castillo et al.

**Supplementary** **Table 5.** Recombination and mutation rates of defense systems Pfams in RSSC.

| **Pfam accessions for defense systems** | **ρ**  **(per site)** | **θ**  **(per site)** | **ρ/θ** |
| --- | --- | --- | --- |
| PF00145 | 0.02618 | 0.05988 | 0.437269 |
| PF00270 | 0.00462 | 0.03755 | 0.12305 |
| PF00580 | 0.00372 | 0.0323 | 0.1151 |
| PF01170 | 0.002893 | 0.05556 | 0.05208 |
| PF01381 | 0.005619 | 0.03339 | 0.168 |
| PF01555 | 0.05305 | 0.07619 | 0.696 |
| PF01844 | 0.009294 | 0.09078 | 0.1023 |
| PF01850 | 0.00006525 | 0.02511 | 0.002598 |
| PF01867 | 0.00707 | 0.04713 | 0.15 |
| PF02384 | 0.003409 | 0.05961 | 0.0572 |
| PF02463 | 0.003523 | 0.03472 | 0.10146 |
| PF02604 | 0.00008058 | 0.05261 | 0.001532 |
| PF02794 | 0.004369 | 0.0497 | 0.08791 |
| PF03364 | 0.001201 | 0.03147 | 0.03815 |
| PF03658 | 0.005248 | 0.06628 | 0.0792 |
| PF03693 | 0.011758 | 0.04784 | 0.2458 |
| PF04014 | 0.00007128 | 0.04716 | 0.001511 |
| PF04221 | 0.00269 | 0.01775 | 0.151548 |
| PF04313 | 0.003454 | 0.06949 | 0.0497 |
| PF04380 | 0.003102 | 0.03805 | 0.081527 |
| PF04471 | 0.01468 | 0.07869 | 0.186 |
| PF04851 | 0.008537 | 0.02647 | 0.32249 |
| PF05016 | 0.00402 | 0.07395 | 0.05436 |
| PF05534 | 0.005521 | 0.04642 | 0.1189 |
| PF05973 | 0.05071 | 0.04258 | 1.191 |
| PF06296 | 0.0001956 | 0.02292 | 0.008537 |
| PF07804 | 0.00122 | 0.04388 | 0.0278 |
| PF08463 | 0.004509 | 0.0709 | 0.06356 |
| PF08798 | 0.02009 | 0.07831 | 0.2564 |
| PF08843 | 0.00003244 | 0.05823 | 0.0005572 |
| PF09344 | 0.01854 | 0.05593 | 0.33148 |
| PF09481 | 0.02661 | 0.06914 | 0.3849 |
| PF09485 | 0.05569 | 0.08265 | 0.6737 |
| PF09660 | 0.00004487 | 0.04748 | 0.0009451 |
| PF09661 | 0.00001 | 0.03899 | 0.0002565 |
| PF09664 | 0.00001 | 0.05373 | 0.0001863 |
| PF09704 | 0.0552 | 0.07026 | 0.7856 |
| PF09707 | 0.00821 | 0.05317 | 0.1544 |
| PF09952 | 0.00173 | 0.0619 | 0.02794 |
| PF11459 | 0.00001209 | 0.02264 | 0.0005342 |
| PF11796 | 0.00001 | 0.05373 | 0.0001863 |
| PF12161 | 0.00323 | 0.05983 | 0.05398 |
| PF13361 | 0.03312 | 0.03215 | 0.103 |
| PF13560 | 0.000041 | 0.05353 | 0.000766 |
| PF13657 | 0.001315 | 0.04323 | 0.03042 |
| PF15738 | 0.001963 | 0.009445 | 0.2078 |
| PF17194 | 0.00001237 | 0.02374 | 0.0005211 |
| PF18019 | 0.00951 | 0.04917 | 0.19339 |
| Average | 0.010 | 0.050 | 0.165 |
| Average for  genomic data*^a^* | 0.0054 | 0.011 | 0.804 |

*^a^* Average values calculated from Table 1, Castillo & Agathos, 2019.

# Supplementary Figures

**Supplementary Figure 1**. Gain and loss of defense system genes in RSSC. Trees were reconstructed with Pfam data for a) Gabija b) Abi c) RM d) CRISPR.

**
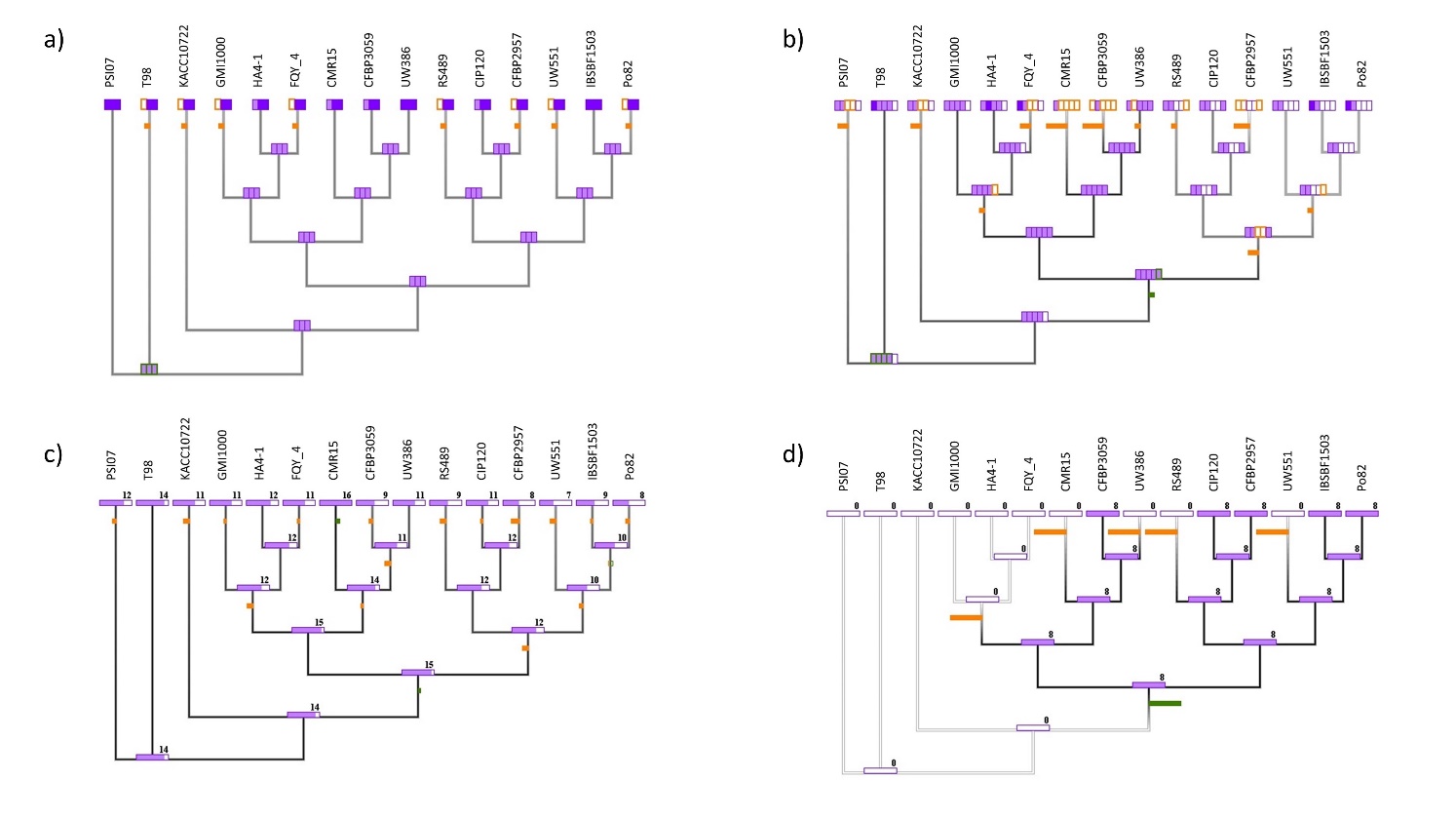
**

**Supplementary Figure** **2.** Summary of Notung results of defense systems in RSSC shown as a network. Nodes represent donor/recipient strains colored by phylotypes. Edges represent HGT events between strains. Edge width represents the number of HGT events between two strains (i.e. the wider the edge, the higher the number of HGT events).

**Argonaute**


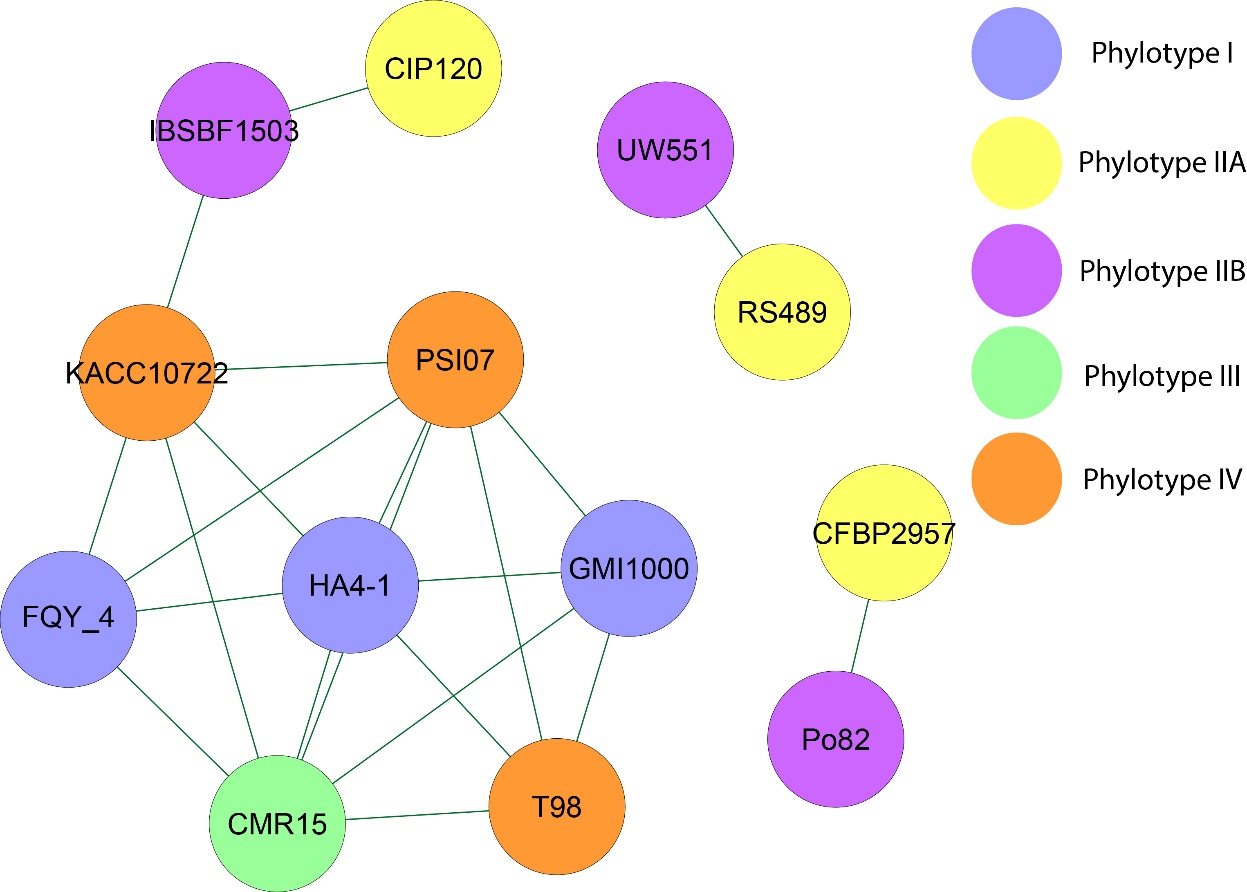


**CRISPR-Cas**


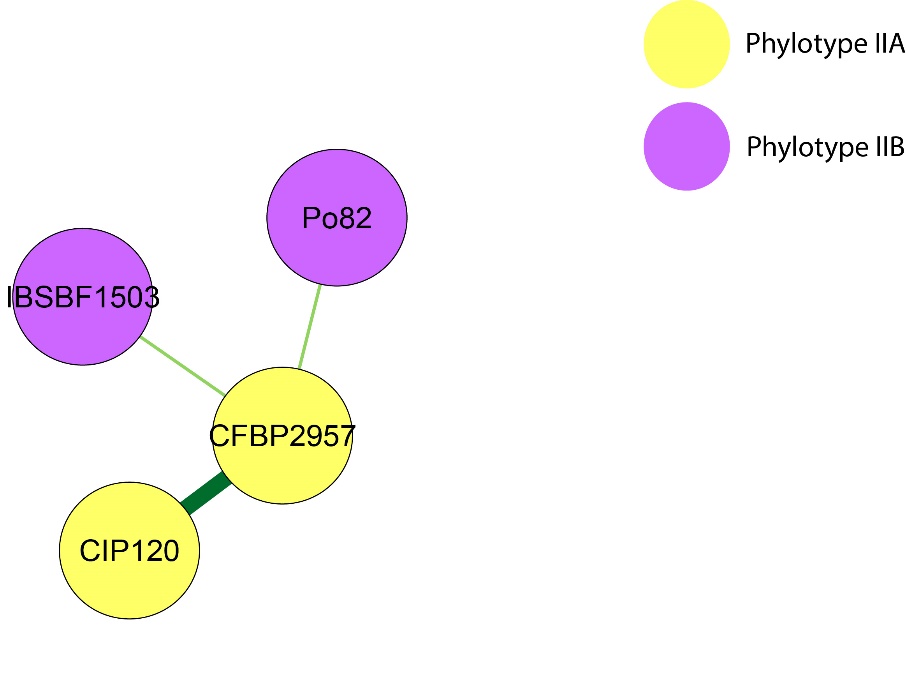


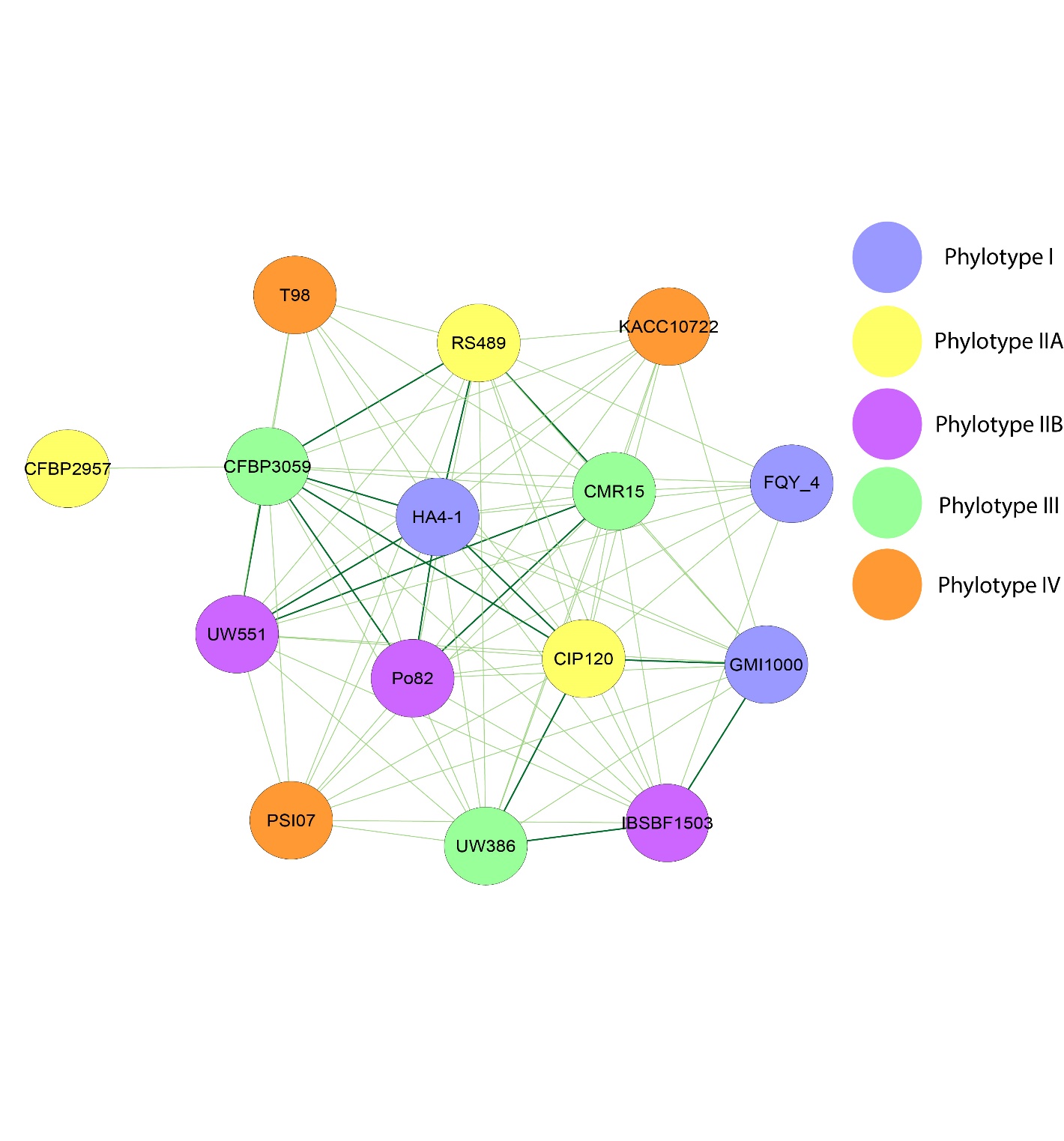
**Gabija**


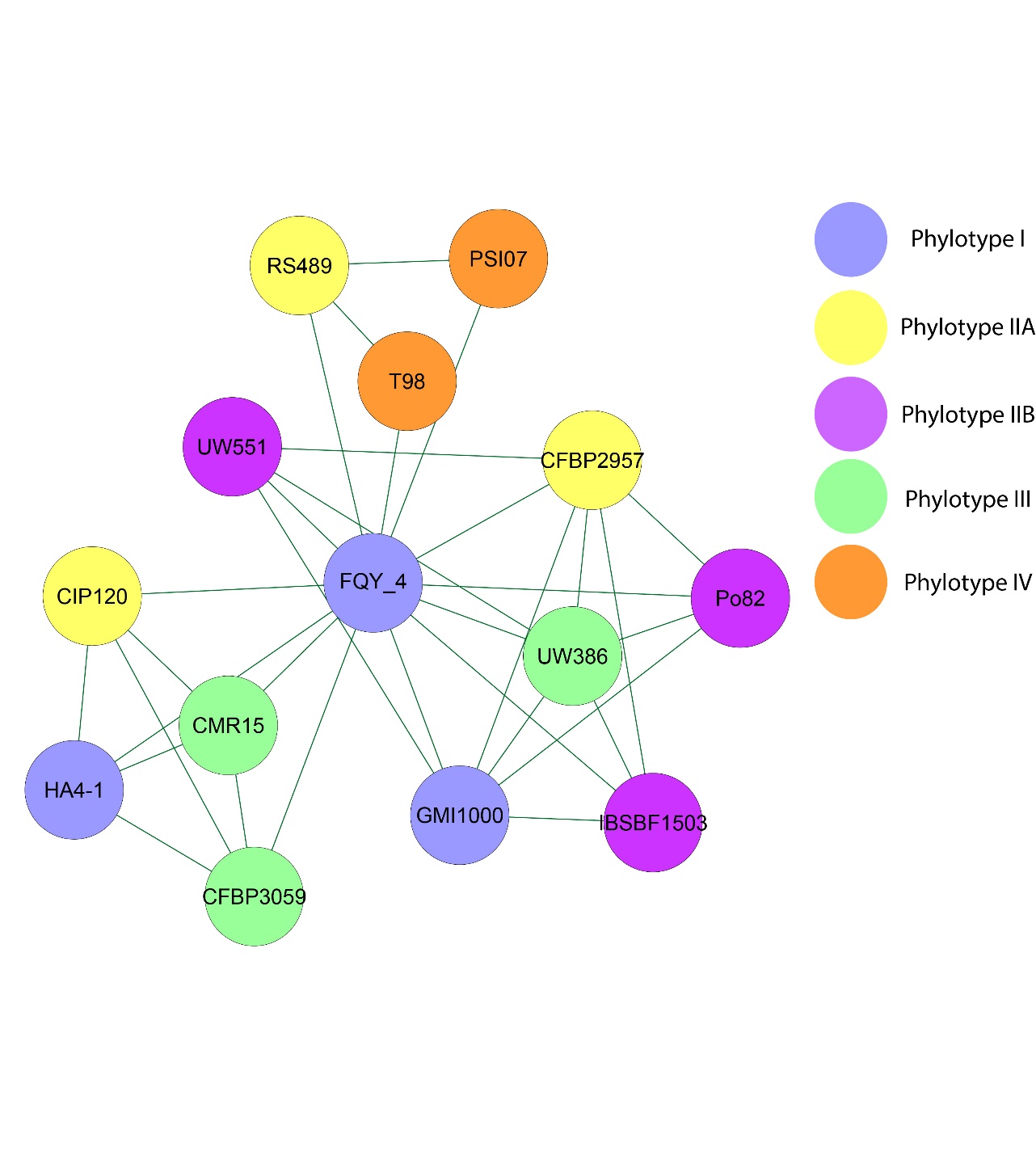
**Hachiman**


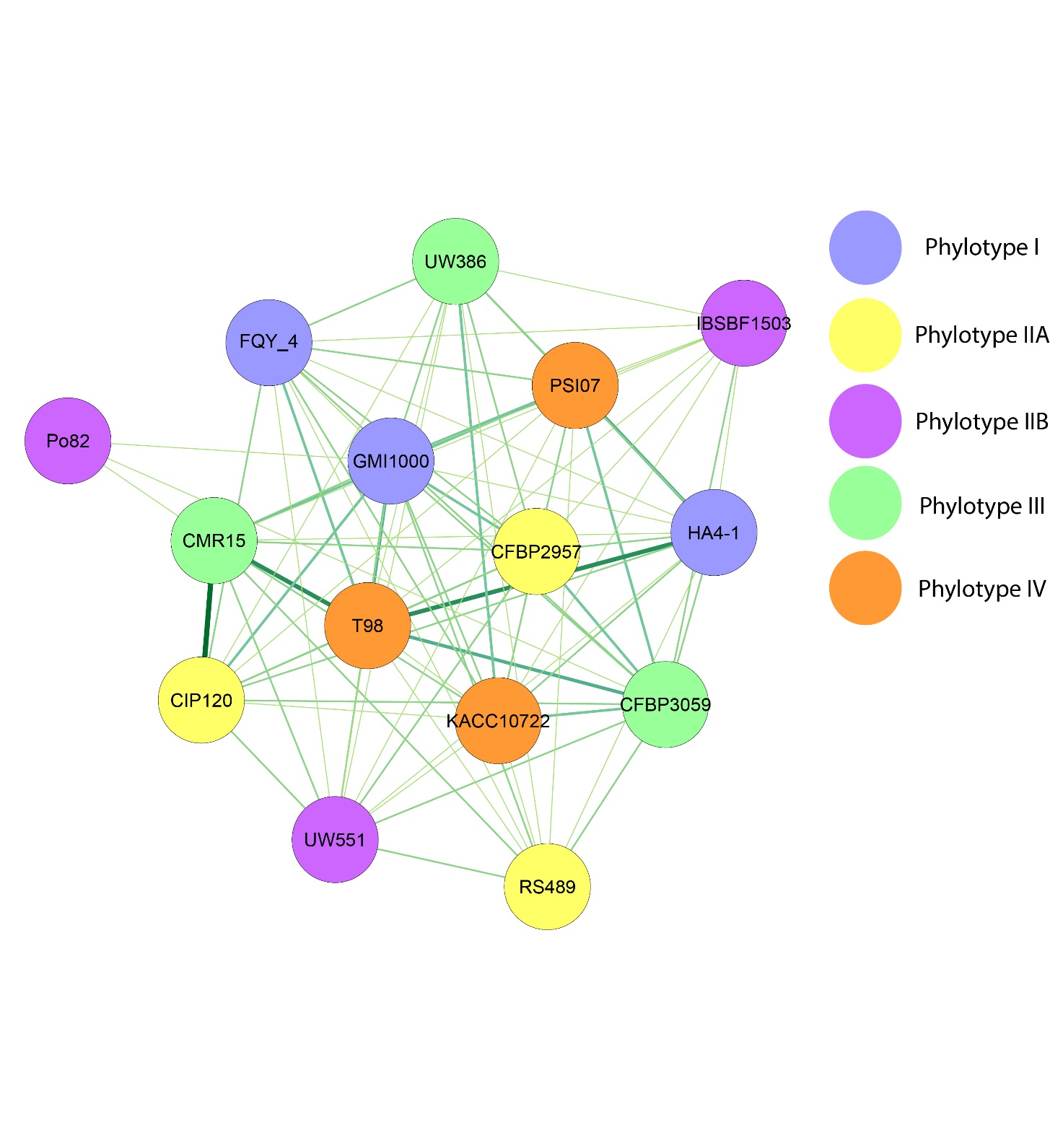
**RM**


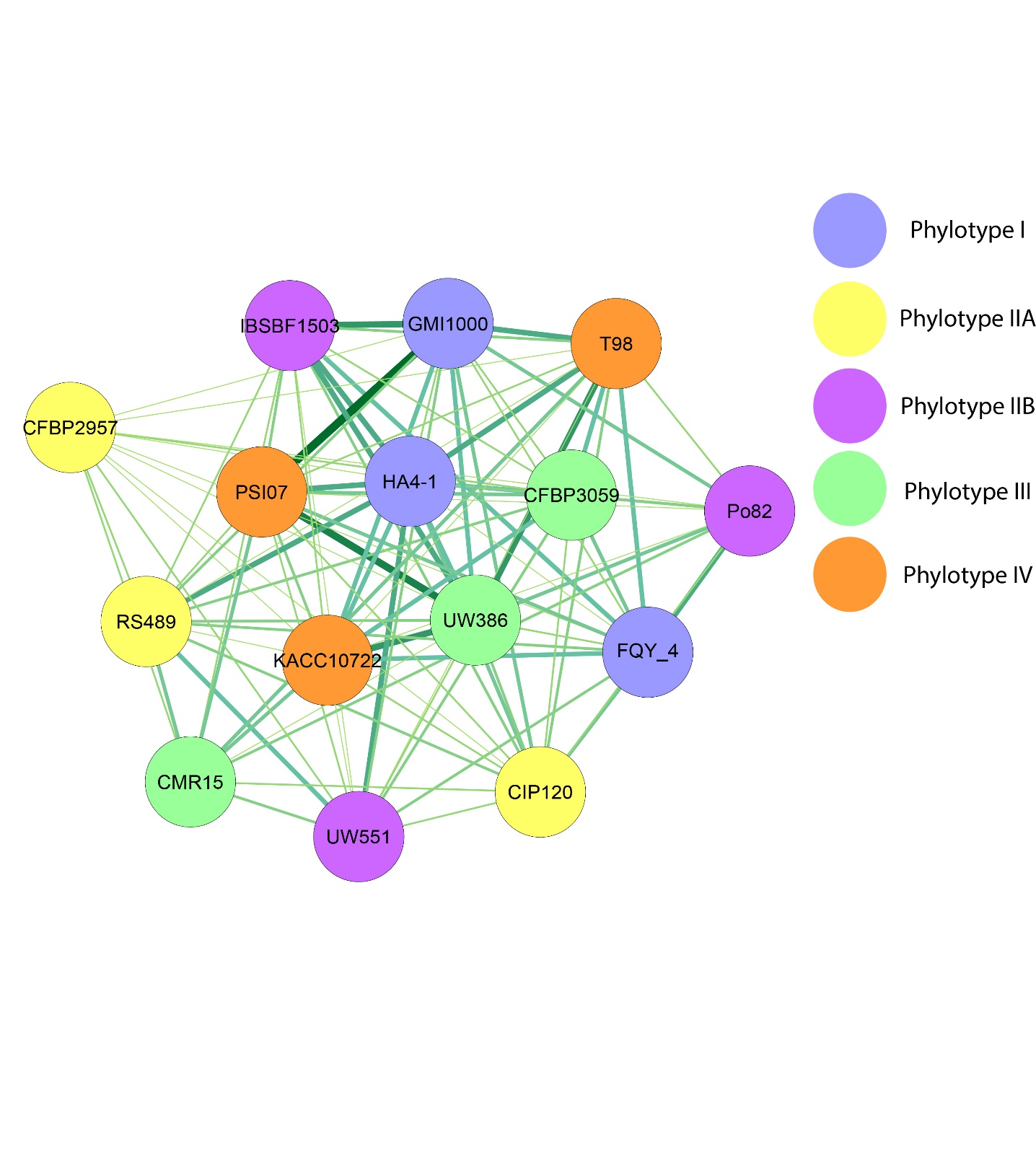
**TA-Abi**

**Thoeris**


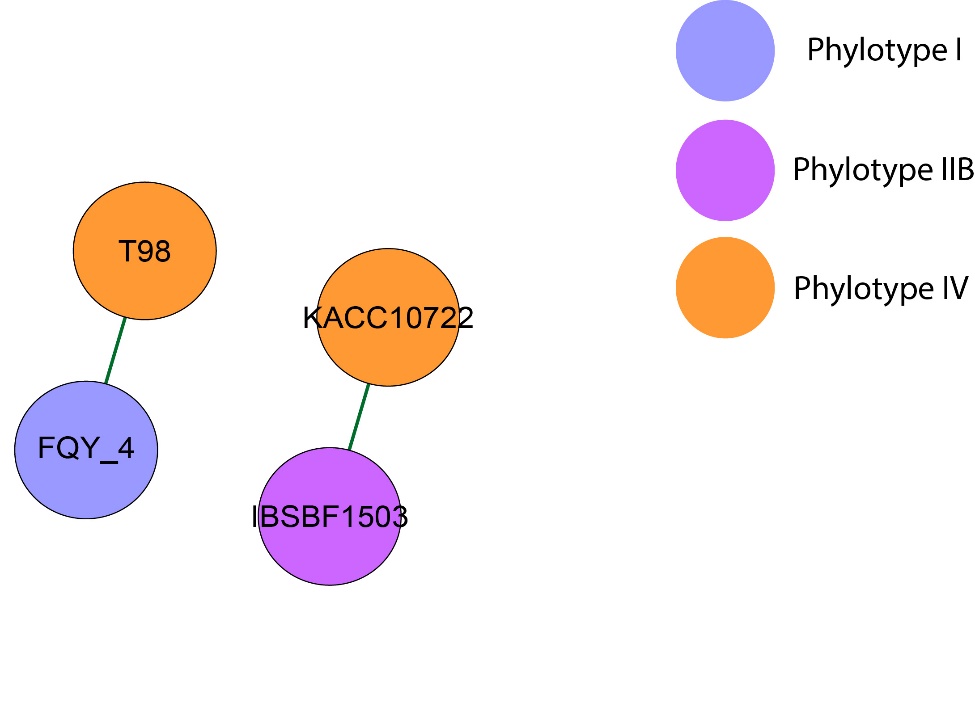


**Wadjet**
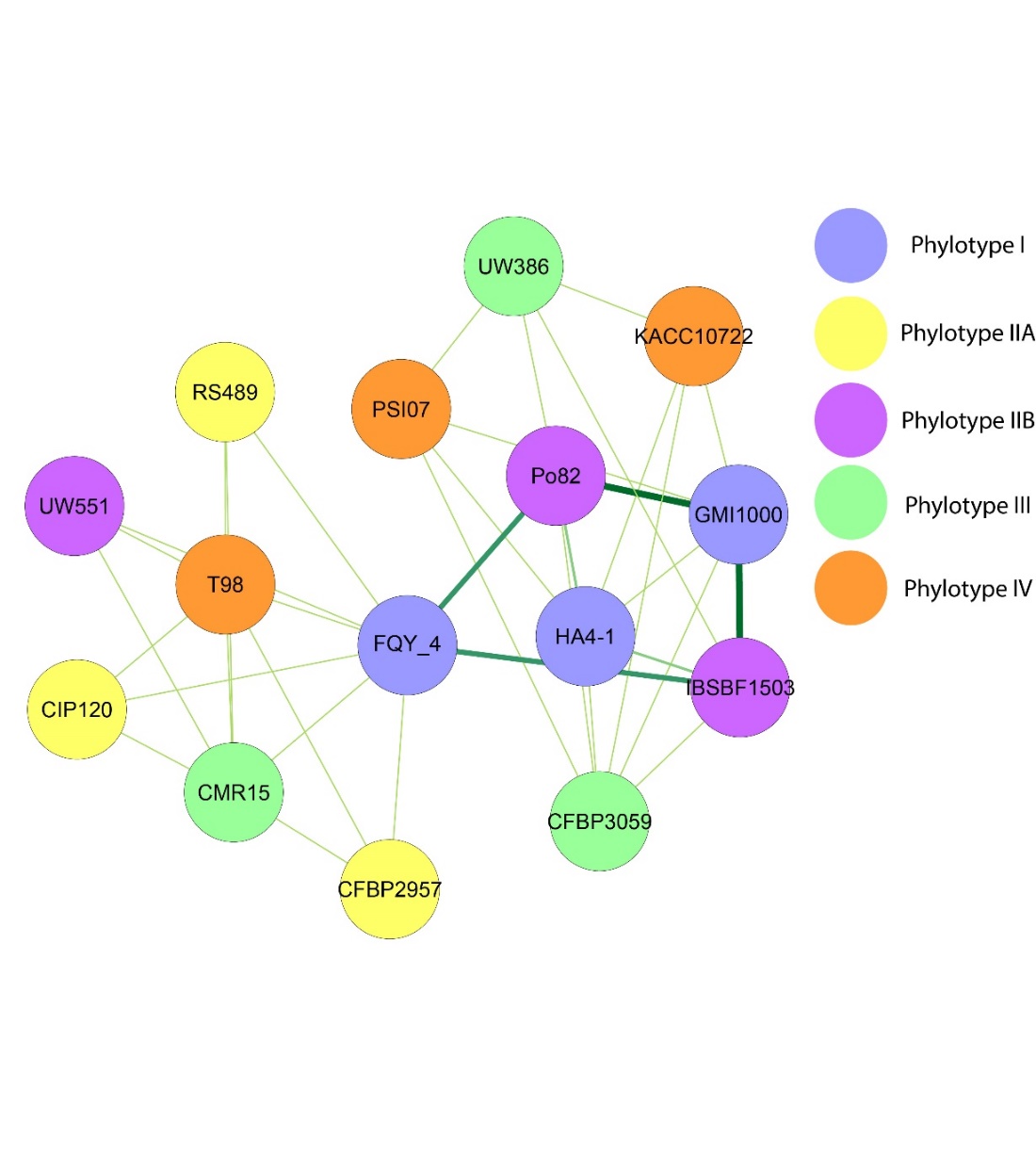

Supplement: Supplementary file 1 [file Data_Sheet_1.docx]
